# Adenosine‐Dependent Arousal Induced by Astrocytes in a Brainstem Circuit

**DOI:** 10.1002/advs.202407706

**Published:** 2024-11-04

**Authors:** Yuwei Zhu, Jiale Ma, Yulan Li, Mengyang Gu, Xiang Feng, Yujin Shao, Lei Tan, Hui‐fang Lou, Li Sun, Yijun Liu, Ling‐hui Zeng, Zilong Qiu, Xiao‐ming Li, Shumin Duan, Yan‐qin Yu

**Affiliations:** ^1^ Department of Neurology of Second Affiliated Hospital and School of Brain Science and Brain Medicine Zhejiang University School of Medicine Hangzhou 310058 China; ^2^ Liangzhu Laboratory MOE Frontier Science Center for Brain Science & Brain‐Machine Integration State Key Laboratory of Brain‐machine Intelligence Zhejiang University Hangzhou 311121 China; ^3^ Nanhu Brain‐computer Interface Institute Hangzhou 311100 China; ^4^ Key Laboratory of Novel Targets and Drug Study for Neural Repair of Zhejiang Province, School of Medicine Hangzhou City University Hangzhou 310015 China; ^5^ Department of Neurology Songjiang Hospital Songjiang Research Institute MOE‐Shanghai Key Laboratory for Children's Environmental Health Shanghai Jiao Tong University School of Medicine Shanghai 200025 China; ^6^ NHC and CAMS Key Laboratory of Medical Neurobiology Zhejiang University Hangzhou 310058 China

**Keywords:** adenosine, arousal, astrocyte, neural circuit, parafacial zone

## Abstract

Astrocytes play a crucial role in regulating sleep‐wake behavior. However, how astrocytes govern a specific sleep‐arousal circuit remains unknown. Here, the authors show that parafacial zone (PZ) astrocytes responded to sleep‐wake cycles with state‐differential Ca^2+^ activity, peaking during transitions from sleep to wakefulness. Using chemogenetic and optogenetic approaches, they find that activating PZ astrocytes elicited and sustained wakefulness by prolonging arousal episodes while impeding transitions from wakefulness to non‐rapid eye movement (NREM) sleep. Activation of PZ astrocytes specially induced the elevation of extracellular adenosine through the ATP hydrolysis pathway but not equilibrative nucleoside transporter (ENT) mediated transportation. Strikingly, the rise in adenosine levels induced arousal by activating A_1_ receptors, suggesting a distinct role for adenosine in the PZ beyond its conventional sleep homeostasis modulation observed in the basal forebrain (BF) and cortex. Moreover, at the circuit level, PZ astrocyte activation induced arousal by suppressing the GABA release from the PZ^GABA^ neurons, which promote NREM sleep and project to the parabrachial nucleus (PB). Thus, their study unveils a distinctive arousal‐promoting effect of astrocytes within the PZ through extracellular adenosine and elucidates the underlying mechanism at the neural circuit level.

## Graphical abstract

This study shows that astrocyte activation in the PZ promotes arousal at the expense of NREM and REM sleep. Adenosine, produced by astrocytes via CD73/39, promotes wakefulness through A_1_ receptors. Additionally, astrocytes suppress GABA release from PZ^GABA^ neurons projecting to the PB, emphasizing astrocyte‐neuron communication in arousal regulation at the circuit level.

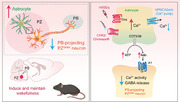

## 1 Introduction

Sleep, as a pervasive behavior observed across species, has conventionally been attributed solely to neuronal regulation within established sleep and arousal centers.^[^ ^1^ ^]^ However, astrocytes, a major type of glial cell within the central nervous system (CNS) and integral components of the tripartite synapse play a crucial role in neuronal network functioning.^[^ ^2^, ^3^ ^]^ They impact sleep‐wake regulation through three distinct pathways.^[^ ^4^ ^]^ First, astrocytes, as essential components of the glymphatic system, participate in waste removal during sleep that may prevent impairments potentially contributing to conditions like Alzheimer's disease.^[^ ^5^ ^]^ Second, astrocytes assume significant metabolic and energy‐supplying functions by providing neurons with lactate via an astrocyte‐neuron shuttle.^[^ ^6^ ^]^ More importantly, astrocytes have been reported to directly modulate sleep homeostasis by releasing sleep‐regulating substances, such as glutamate,^[^ ^7^ ^]^ ATP,^[^ ^8^ ^]^ and adenosine,^[^ ^9^ ^]^ thereby promoting sleep drive. However, inconsistent findings have been reported regarding the exact roles of astrocytic activities in sleep‐arousal regulation. Astrocytic Ca^2+^ activity has been found to fluctuate in parallel with sleep‐wake transitions, exhibiting varying patterns across different brain regions.^[^ ^10^, ^11^, ^12^ ^]^ The same brain region has shown incongruent astrocytic Ca^2+^ activities, likely due to differences in Ca^2+^ recording sensors’ sensitivity and the characteristics of various optical imaging approaches.^[^ ^12^, ^13^ ^]^ Moreover, astrocytes have been identified exerting divergent regulatory roles in various brain regions, such as regulating sleep need in the cortex,^[^ ^10^, ^14^ ^]^ promoting wakefulness in the lateral hypothalamus (LH),^[^ ^15^ ^]^ and enhancing sleep in the ventrolateral preoptic area (VLPO).^[^ ^16^, ^17^ ^]^ Therefore, despite considerable research efforts has been devoted to the study of astrocytes, our understanding of their activities and how they contribute to the functions of sleep circuits and behavior remains comparatively primitive.

The parafacial zone (PZ) is a delimited cluster of medullary neurons situated laterally and dorsally to the facial nerve (7n). It is acknowledged as a critical non‐rapid eye movement (NREM) sleep‐promoting center within the brainstem.^[^ ^18^, ^19^ ^]^ Prior research indicates that the chemogenetic activation of GABAergic neurons in the PZ increases NREM sleep at the expense of both wakefulness and rapid eye movement (REM) sleep.^[^ ^19^ ^]^ This effect occurs through a proposed neural circuit with projection from PZ^GABA^ to parabrachial (PB) neurons, which subsequently project and release glutamate onto cortically projecting neurons in the magnocellular basal forebrain (BF).^[^ ^19^ ^]^ While previous efforts to comprehend the sleep‐regulating role of the PZ have primarily focused on neuronal mechanisms,^[^ ^20^, ^21^ ^]^ the precise functions and chemical mechanisms of astrocytes in sleep‐wake cycles at the circuit level remain largely unexplored. Thus, we employed the PZ as an illustrative model to investigate the involvement of astrocytes in sleep‐wake related circuit, which not only promises to yield insights into the mechanisms governing sleep‐wake regulation but also facilitates the synthesis of overarching principles governing intercellular communication throughout the brain, a process critical for animal behavior regulation.

Over several years, our laboratory has amassed a substantial body of research in the field of glia‐neuron interconnectivity and interactions.^[^ ^22^, ^23^, ^24^ ^]^ This study seeks to elucidate the unique role of astrocytes within the PZ region in regulating sleep‐wake cycles. To validate our hypothesis, we monitored astrocytic activities using fiber photometry and employed optogenetic and chemogenetic techniques to manipulate astrocytes in freely moving mice. Additionally, we delved into the underlying chemical and neurological mechanisms through a combination of optogenetics, microinjection, and fiber photometry recordings of Ca^2+^, ATP, and adenosine signaling. Our study unveils a distinctive arousal‐promoting effect of astrocytes within the PZ, mediated by adenosine, and elucidates the underlying mechanism at the neural circuit from the NREM sleep‐promoting PZ^GABA^ neurons projecting to the PB.

## 2 Results

### 2.1 Astrocytic Ca^2+^ Activity in the PZ Rises with Arousal

**Figure 1:**
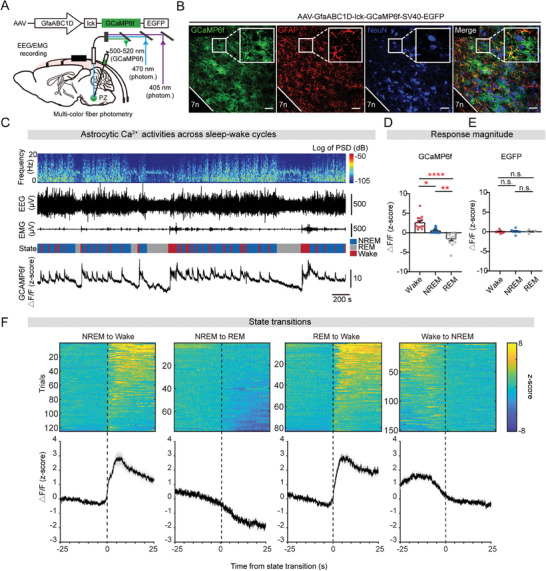
Elevated astrocytic Ca^2+^ activity in the PZ during wakefulness. A) Setup for fiber photometric recording in the PZ astrocytes during sleep‐wake cycles in combination with EEG/EMG recordings. B) Representative images of AAV‐GfaABC1D‐GCaMP6f‐EYFP expression in the PZ (position of coronal section) co‐localized with GFAP, while not with NeuN. Scale bar, 50 µm; green, GCaMP6f ‐EYFP; red, GFAP; blue, NeuN. C) Top to bottom, EEG power spectrogram (0–20 Hz), EEG traces, EMG traces, vigilant states (blue, NREM sleep; grey, REM sleep; red, wakefulness), and astrocytic Ca^2+^ activity (z‐score). D) Mean ΔF/F (z‐score) of GCaMP6f during wakefulness, NREM sleep, and REM sleep. *n =* 14 mice, a Kruskal–Wallis test, Dunn's multiple comparisons test; Wake versus NREM *p = 0.0242, Wake versus REM ^****^ *p* < 0.0001, NREM versus REM ^**^ *p* = 0.0076. E) Mean ΔF/F (z‐score) of EGFP during wakefulness, NREM sleep, and REM sleep. *n =* 10 mice, a one‐way ANOVA test, Tukey's multiple comparisons test, n.s. indicates not statistically significant. F) Heatmaps of Ca^2+^ fluorescence traces during the 25 s before and after state transitions between NREM sleep, REM sleep, and wakefulness (top). Line plots are mean ΔF/F (±s.e.m.) during state transitions under baseline conditions (bottom). Vertical dashed lines indicate time of state transitions. *n =* 14 mice.

To explore the involvement of PZ astrocytes in sleep‐wake behavior, we initially investigated the intracellular Ca^2+^ activities of astrocytes throughout sleep‐wake cycles. We injected an adeno‐associated virus (AAV) encoding GCaMP6f protein (AAV2/5‐GfaABC1D‐lck‐GCaMP6f‐SV40‐EGFP) into the PZ and employed multicolor fiber photometry to minimize the influence of movement artifacts (**Figure** **1**A). To confirm the specificity of GCaMP6f expression in astrocytes, we performed post hoc immunostaining and found that GCaMP6f colocalized with the glial fibrillary acidic protein (GFAP) and not with the neuronal nuclei (NeuN) (Figure 1B). Additionally, staining with the astrocyte marker S100 calcium binding protein beta (S100β) further verified the high specificity expression of GCaMP6f expression, with 98.98% GCaMP6f‐positive cells overlapping with S100β and only 1.012% overlapping with NeuN (Figure S1A,B, Supporting Information). We also checked the range of GCaMP6f expression in the PZ (Figure S2A, Supporting Information). According to unintermittent fiber photometric recordings, we found that astrocytic Ca^2+^ activity discernibly peaks during wakefulness, while gradually decreased during NREM and REM sleep (Figure 1C). We then analyzed the mean GCaMP6f fluorescence change (ΔF/F, z‐score; see Methods for details) in each vigilant state, and we found that astrocytic Ca^2+^ activity was highest during wakefulness and lowest during REM sleep, whereas the mean EGFP fluorescence as control remained relatively stable during sleep‐wake cycles (Figure 1D,E; Figure S3, Supporting Information). Notably, transitions from NREM or REM sleep to wakefulness were marked by a significant increase in astrocytic Ca^2+^ activity, whereas the reverse was observed during transitions from NREM to REM sleep or from wakefulness to NREM sleep (Figure 1F). Taken together, our results suggest that PZ astrocytes exhibit state‐dependent Ca^2+^ activities that surge accompanied by transitions from sleep to wakefulness.

### 2.2 Activation of PZ Astrocytes Drives and Maintains a Region‐Dependent Arousal

**Figure 2:**
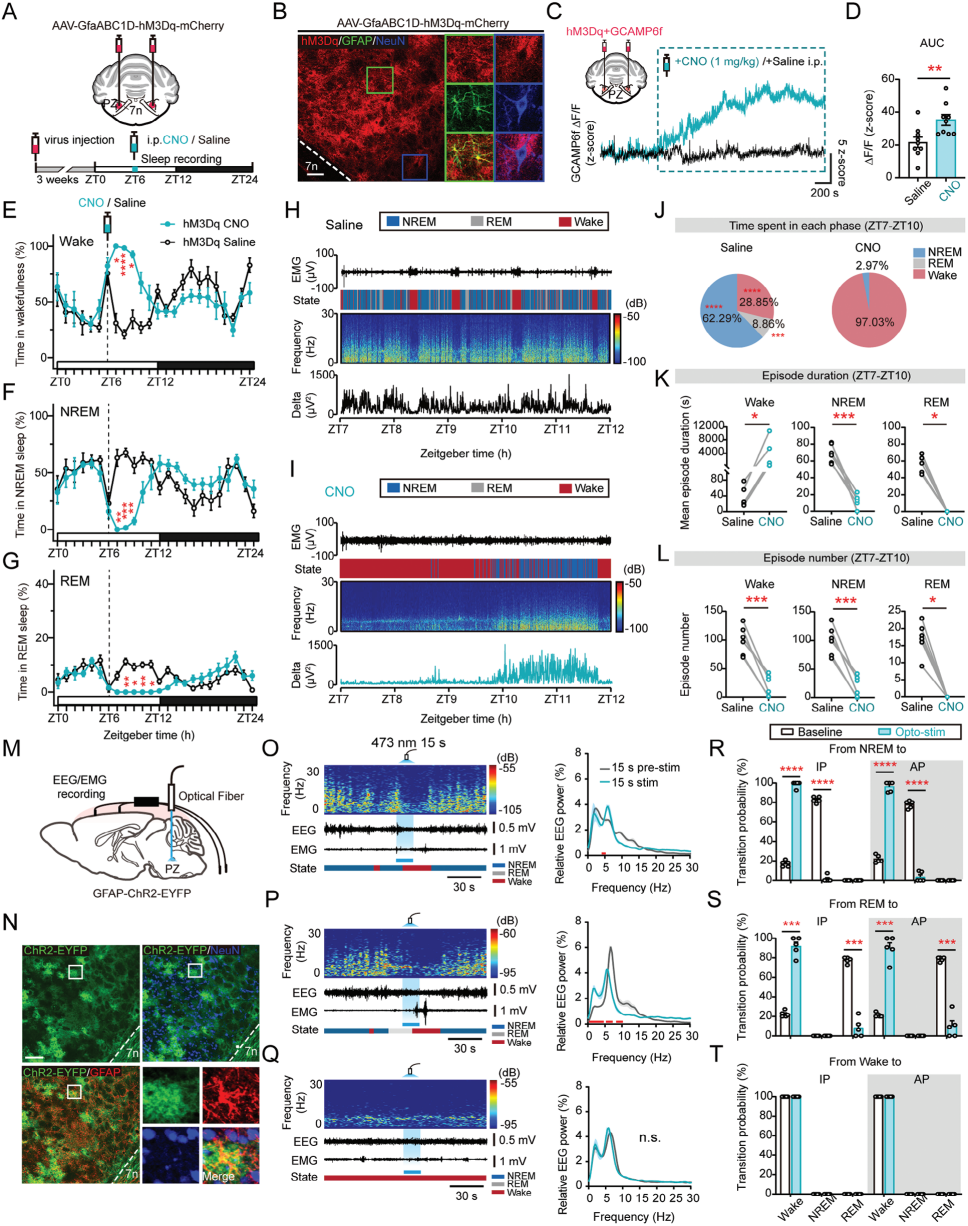
Chemogenetic and optogenetic activation of PZ astrocytes induces wakefulness. A) Setup for chemogenetic activation of astrocytes within the PZ via bilateral injection of AAV‐GfaABC1D‐hM3Dq‐mCherry virus. B) Representative images of AAV‐GfaABC1D‐hM3Dq‐mCherry expression within the PZ (position of coronal section indicated by dashed line in schematic) co‐localized with GFAP, while not with NeuN. Scale bar, 50 µm; red, hM3Dq‐mCherry; green, GFAP; blue, NeuN. C) Setup for bilateral fiber photometric recording of astrocytic Ca^2+^ activity within the PZ by mixed injection of GCaMP6f with hM3Dq virus (upper left). Representative Ca^2+^ fluorescent traces (z‐score) during the pre‐ and post‐injection of CNO (1 mg kg^−1^) or saline intraperitoneally (bottom). D) Area under curve (AUC) of astrocytic Ca^2+^ activity (z‐score) between CNO and saline group. *n =* 9 trials recorded from 3 mice, an unpaired two‐tailed *t*‐test, ^**^ *p* = 0.0093. E–G) Hourly percentages (±s.e.m.) of wakefulness (E), NREM sleep (F), and REM sleep (G) of saline (*n =* 6 mice) and CNO group (*n =* 6 mice) during ZT0‐ZT24. Two‐way ANOVA test, Sidak's multiple comparisons test, Wake: ^*^ *p* = 0.0122 (ZT7), ^****^ *p* < 0.0001 (ZT8), ^*^ *p* = 0.0107 (ZT9); NREM: ^**^ *p* = 0.0088 (ZT7), ^***^ *p* = 0.0001 (ZT8), ^**^ *p* = 0.0090 (ZT9); REM: ^**^ *p* = 0.0053 (ZT8), ^*^ *p* = 0.0203 (ZT9), ^**^ *p* = 0.0041 (ZT10), ^*^ *p* = 0.0160 (ZT11). H,I) Representative graph of state changes in 5 h after saline (H) or CNO (I) injection. Top to bottom, EMG traces, vigilant states (color coded), EEG power spectrogram (0‐30 Hz), and delta wave power (µV^2^). J) Total time spent in each state during ZT7‐ZT10 between saline (*n =* 6 mice) and CNO group (*n =* 6 mice). Two‐way ANOVA test, Sidak's multiple comparisons test, ^****^ *p* < 0.0001 (Wake), ^***^ *p* = 0.0004 (REM), ^****^ *p* < 0.0001 (NREM). K) Mean episode duration of each state in 3 h after CNO or saline injection (ZT7‐ZT10). Each pair of dots, data from one mouse (saline, *n =* 6 mice; CNO, *n =* 6 mice). Wake: a two‐tailed Wilcoxon rank test, ^*^ *p* = 0.0313; NREM: a two‐tailed paired t test, ^***^ *p* = 0.0003; REM: a two‐tailed Wilcoxon rank test, ^*^ *p* = 0.0313.L) Episode number of each state in 3 h after CNO or saline injection (ZT7‐ZT10). Saline, *n =* 6 mice; CNO, *n =* 6 mice. Wake: a two‐tailed paired t test, ^***^ *p* = 0.0003; NREM: a two‐tailed paired t test, ^***^ *p* = 0.0003; REM: a two‐tailed Wilcoxon rank test, ^*^ *p* = 0.0313. M) Setup for optogenetic activation of astrocytes by embedding optical fiber on top of PZ in GFAP‐ChR2‐EYFP rats. N) Representative images of ChR2‐EYFP expression in the PZ (position of coronal section indicated by dashed line in schematic) co‐localized with GFAP, while not with NeuN. Scale bar, 100 µm; green, ChR2‐EYFP; red, GFAP; blue, NeuN. O–Q) Left, example graphs of optogenetic activation of PZ astrocytes respectively in NREM sleep (O), REM sleep (P), and wakefulness (Q). Top to bottom, brain states (color coded), EEG power spectrogram (0–30 Hz), EEG, and EMG traces. Light blue shading indicates 15 s blue laser stimulation. Right, comparison of relative EEG power (±s.e.m.) of 0–30 Hz between 15 s pre‐ and during stimulation. Red line indicates statistically significant. Two‐way ANOVA test, Bonferroni's multiple comparisons test. R–T) Probability of state transitions with and without optogenetic activation of astrocytes. *n =* 5 rats. Two‐way ANOVA test, Sidak's multiple comparisons test, IP: ^***^ *p* = 0.0006 (REM to Wake), ^***^ *p* = 0.0006 (REM to REM); AP: ^***^ *p* = 0.0005 (REM to Wake), ^***^ *p* = 0.0005 (REM to REM), ^****^ *p* < 0.0001.

We then asked whether astrocyte activation per se is sufficient to elicit sleep‐wake behavior. hM3Dq‐encoding AAV (AAV2/5‐GfaABC1D‐hM3Dq‐mCherry) bilaterally injected mice were intraperitoneally (i.p.) injected with clozapine‐N‐oxide (CNO, 1 mg kg^−1^) at ZT6 to chemogenetically activate the PZ astrocytes (**Figure** **2**A). The time point for chemogenetic activation was chosen because mice exhibit the most sleep at ZT6 during the 24‐h cycle, when arousal levels are lowest. This allows us to better assess the arousal‐promoting role of activation by minimizing the influence of basal vigilance. hM3Dq expression was confirmed in GFAP‐positive astrocytes by post hoc immunostaining of GFAP and NeuN (Figure 2B). We also checked the range of hM3Dq expression in the PZ (Figure S2B, Supporting Information). CNO treatment robustly induced an elevation of astrocytic Ca^2+^ activity (Figure 2C,D). CNO administration elicited consistent arousal in the first 3 h following CNO injection (ZT7‐10) (Figure 2E–J). The sudden increased wakefulness observed at ZT6 resulted from an intraperitoneal injection procedure (Figure 2E). Interestingly, both NREM and REM sleep were almost completely absent (Figure 2F–I). Moreover, after the subsiding of the CNO effect, there was a compensatory restoration of NREM and REM sleep (Figure 2F–I). Notably, we found that the mean duration of wakefulness episodes was significantly extended after CNO injection at the expense of both NREM and REM sleep (Figure 2K), and the number of NREM and REM sleep episodes is significantly reduced (Figure 2L), indicating a critical role of astrocytes in maintaining arousal state. The dosage of CNO injected was confirmed with no impact on sleep‐wake cycles (Figure S4, Supporting Information).

To examine the direct relationship between PZ astrocytes and sleep‐wake behavior with a more precise temporal resolution, we developed astrocyte‐targeted ChR2‐expressed rats (GFAP‐ChR2‐EYFP), which also enabled us to evaluate the applicability of the sleep‐regulatory effect of PZ astrocytes across different rodent species (Figure 2M). ChR2 facilitates intracellular Ca^2+^ elevation within astrocytes through reversing the Na^+^‐Ca^2+^ exchanger following Na^+^ influx through ChR2 channels, allowing for more temporally precise control.^[^ ^25^, ^26^ ^]^ The specificity of ChR2‐EYFP expression was first validated in vivo in GFAP‐positive astrocytes by post hoc immunostaining (Figure 2N). We also stained with S100β which showed highly specific expression of ChR2 with 99.56% overlapping and only 0.44% overlapping with cells expressing NeuN (Figure S1C,D, Supporting Information). It was further confirmed in vitro by culturing and immunostaining cells from the brainstem and PZ of GFAP‐ChR2‐EYFP rats (Figure S1E,F, Supporting Information). The effectivity of astrocyte response to optogenetic activation has been previously confirmed by whole‐cell recording in our lab.^[^ ^27^ ^]^ Notably, we found that optogenetic stimulation (473 nm, 5 mW, 15 s, direct current) evoked rapid transitions to wakefulness from both NREM and REM sleep (Figure 2O–Q). Moreover, we explored whether the arousal‐inducing effect of astrocytes varies with the circadian rhythm. It is noteworthy that the probability of transitions between different states remained consistent between the inactive period (IP) and the active period (AP) (Figure 2R–T). In summary, PZ astrocytes were found to play a crucial role in initiating and sustaining arousal, revealing an additional facet of the PZ beyond its known role as a sleep‐promoting center.

Furthermore, it is worth noting that various types of astrocytic activities during sleep‐wake cycles have been documented in different brain regions.^[^ ^12^ ^]^ In addition to investigating astrocytic roles in the brain stem, we explored their roles in other brain regions, including the BF and LH. We unilaterally injected astrocyte‐specific AAV encoding ChrimsonR (AAV2/5‐GfaABC1D‐ChrimsonR‐mCherry) into the PZ, BF, or LH of mice respectively, and optogenetically stimulated (635 nm, 5 mW, 15 s, direct current) in different vigilant states (Figure S5A, Supporting Information). Interestingly, astrocyte activation yielded distinct sleep‐regulating effects in the PZ, BF, and LH, that its activation has promoted arousal in the PZ and LH, but having no remarkable impact on sleep‐wake regulation in the BF (Figure 2; Figure S5B,C, Supporting Information).

### 2.3 Astrocyte Ca^2+^ Activity is Required for Sustaining Wakefulness

**Figure 3:**
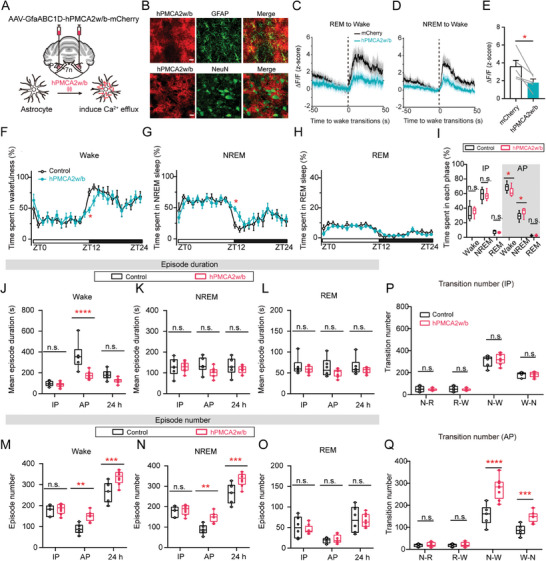
Inhibition of astrocytic Ca^2+^ activities in the PZ with hPMCA2w/b reduced wakefulness. A) hPMCA2w/b bilateral injection to attenuate astrocytic Ca^2+^ activity in the PZ mediated by continuous calcium pumping. B) Representative images of AAV‐GfaABC1D‐hPMCA2w/b‐mCherry expression in the PZ labeling with GFAP (upper) and NeuN (down). Scale bar, 20 µm; red, hPMCA2w/b; green, GFAP or NeuN. C, D) Mean (±s.e.m.) ΔF/F (z‐score) of astrocytic Ca^2+^ activity during the 50 s before and after the transition from REM sleep (C) or NREM sleep (D) to wakefulness between hPMCA2w/b and mCherry group. *n =* 5 mice. E) Mean ΔF/F (z‐score) of astrocytic Ca^2+^ activity between hPMCA2w/b and mCherry group during wakefulness. *n =* 5 mice, paired two‐tailed t‐test, ^*^ *p* = 0.0363. F–H) Hourly percentages (±s.e.m.) of time spent in wakefulness (F), NREM sleep (G), and REM sleep (H) of mice that received mCherry virus injections into PZ (control, *n =* 8 mice), and littermates that received hPMCA2w/b virus injections (hPMCA2w/b, *n =* 10 mice). Two‐way ANOVA test, Sidak's multiple comparisons test, ^*^ *p* = 0.0238 (NREM), ^*^ *p* = 0.0287 (wake). I) Percentages of total time spent in each state during IP and AP. (control, *n =* 9 mice; hPMCA2w/b, *n =* 11 mice). Two‐way ANOVA test, Sidak's multiple comparisons test, ^*^ *p* = 0.0418 (wake), ^*^ *p* = 0.0347 (NREM). J–L) Mean episode duration spent in wakefulness (J), NREM sleep (K), and REM sleep (L) between control and hPMCA2w/b group during IP, AP, and 24h. (control, *n =* 8 mice; hPMCA2w/b, *n =* 10 mice) Two‐way ANOVA test, Sidak's multiple comparisons test, ^****^ *p* < 0.0001. M–O) Episode number of wakefulness (M), NREM sleep (N), and REM sleep (O) between control and hPMCA2w/b group during IP, AP, and 24h. (control, *n =* 8 mice; hPMCA2w/b, *n =* 10 mice) Two‐way ANOVA test, Sidak's multiple comparisons test, Wake, ^**^ *p* = 0.0015, ^***^ *p* = 0.0004; NREM, ^**^ *p* = 0.0016, ^***^ *p* = 0.0004. P,Q) Number of state transitions including NREM sleep to REM sleep (N‐R), REM sleep to wakefulness (R‐W), NREM sleep to wakefulness (N‐W), and wakefulness to NREM sleep (W‐N) between control and hPMCA2w/b group during IP (P) and AP (Q). (control, *n =* 6 mice; hPMCA2w/b, *n =* 8 mice) Two‐way ANOVA test, Sidak's multiple comparisons test, ^***^ *p* = 0.0005, ^****^ *p* < 0.0001.

We then asked whether astrocytic Ca^2+^ activity is necessary for natural sleep‐wake behaviors. We injected an AAV encoding Ca^2+^ pump hPMCA2w/b (AAV2/5‐GfaABC1D‐hPMCA2w/b‐mCherry) into the PZ, which can effectively exclude cytosolic Ca^2+^ from astrocytes, spanning soma, processes, and territories, thereby reducing its global activity (**Figure** **3**A).^[^ ^28^ ^]^ The specificity of hPMCA2w/b expression was confirmed by its colocalization with GFAP, but not with NeuN (Figure 3B). We also assessed the range of hPMCA2w/b expression in the PZ (Figure S2C, Supporting Information). We found that hPMCA2w/b expression robustly reduced astrocytic Ca^2+^ activity during REM‐to‐Wake transitions (Figure 3C) and NREM‐to‐Wake transitions (Figure 3C–E). Strikingly, a remarkable reduction of time in wakefulness was observed during AP, accompanied with an increase in NREM sleep (Figure 3F–I).

To delve into the specific role of astrocytes in regulating sleep structure and quality, we further analyzed the changes in mean episode duration and episode number. Notably, the mean duration of wakefulness episodes was substantially decreased during the AP (Figure 3J–L), while the number of NREM sleep and wakefulness episodes remarkably increased (Figure 3M–O). Moreover, we observed a marked surge in the transitions between NREM sleep and wakefulness during the AP (Figure 3P,Q). To summarize, PZ astrocytes are necessary for sustaining wakefulness mainly by impeding the transition from wakefulness to sleep.

### 2.4 Adenosine Waltzes with Astrocyte Ca^2+^ Activity Throughout Sleep‐Wake Cycles

**Figure 4:**
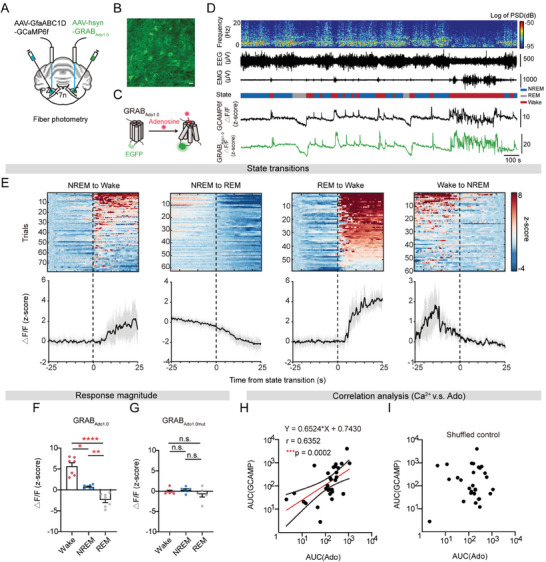
Adenosine dances with astrocytic Ca^2+^ activity in the PZ during sleep‐wake cycles. A) Setup for bilateral fiber photometric recording of adenosine with GRAB_Ado1.0_ and astrocytic Ca^2+^ activity within the PZ during sleep‐wake cycles. B) Representative image of GRAB_Ado1.0_ expression within the PZ. Scale bar, 100 µm; green, GRAB_Ado1.0_. C) Schematic illustrating the principle of GRAB_Ado1.0_ sensors: adenosine binding induces a conformational change thereby elevating EGFP fluorescence. D) Top to bottom, EEG power spectrogram (0‐20 Hz), EEG traces, EMG traces, vigilant states (color coded), and representative astrocytic Ca^2+^ activity and GRAB_Ado1.0_ fluorescence traces (z‐score) during sleep‐wake cycles. E) Heatmaps of GRAB_Ado1.0_ fluorescence traces during the 25 s before and after transitions between NREM sleep, REM sleep, and wakefulness (top). Line plots are mean ΔF/F (±s.e.m.) during state transitions under baseline conditions (bottom). Vertical, dashed lines indicate time of state transition. *n =* 7 mice. F, G) Mean (±s.e.m.) ΔF/F (z‐score) of GRAB_Ado1.0_ (F) and GRAB_Ado1.0mut_ (G) during each state. GRAB_Ado1.0_, *n =* 8 mice; GRAB_Ado1.0mut_, *n =* 6 mice. F, a one‐way ANOVA test, Tukey's multiple comparisons test, ^*^ *p* = 0.0259, ^**^ *p* = 0.0018, ^****^ *p* < 0.0001. G, Kruskal‐Wallis test, Dunn's multiple comparisons test. n.s. indicates not statistically significant. H, I) Correlation analysis of GCaMP6f and GRAB_Ado1.0_ signals (H) and the shuffled control (I). *n =* 29 trials, recorded from 7 individuals. Pearson correlation analysis, ^***^ *p* = 0.0002, r = 0.6352, red line shows linear regression line (Y = 0.6524*X + 0.7430), dot line indicates 95% regression range.

To identify the molecular mediator responsible for evoking and sustaining wakefulness, we examined extracellular gliotransmitter in the PZ across sleep‐wake cycles. Our focus centered on extracellular adenosine, a molecule potentially generated by activated astrocytes, which stems from the ongoing debate regarding adenosine's role in sleep‐wake regulation beyond its established reputation as an endogenous homeostatic sleep factor. We injected AAV encoding GRAB_Ado1.0_ (AAV‐hsyn‐GRABAdo1.0), a genetically encoded G protein‐coupled receptor activation‐based sensor, into one side of the PZ to measure adenosine levels in the extracellular space, while astrocyte‐specific GCaMP6f was injected into the other side to simultaneously record adenosine levels and astrocytic Ca^2+^ activity during sleep‐wake cycles (**Figure** **4**A–C). We confirmed GRAB_Ado1.0_ expression in the PZ by observing robust fluorescence at the cell membrane (Figure 4B). Interestingly, we observed a similar surge in adenosine levels following transitions from NREM or REM sleep to wakefulness (Figure 4D,E), which closely mirrors the dynamics of astrocytic Ca^2+^ activity during the sleep‐wake cycles. Furthermore, adenosine reached its peak during wakefulness and dipped to its lowest levels during REM sleep (Figure 4F). As a comparison, the fluorescence levels of GRAB_Ado1.0mut_, a non‐binding mutant version of GRAB_Ado1.0_, remained relatively stable across different sleep‐wake states (Figure 4G; Figure S6, Supporting Information). We further explored the interrelationship between astrocytic Ca^2+^ activity and adenosine dynamics throughout sleep‐wake cycles. Strikingly, a robust correlation between these two signals was identified (r = 0.6352, Figure 4H). Such correlation was not observed in the control analysis, in which the GRAB_Ado1.0_ signals were temporally shuffled in a random manner (r = 0.06822, Figure 4I). Collectively, these findings provide evidence linking the increase in astrocytic Ca^2+^ activity during wakefulness to the upsurge of extracellular adenosine in the PZ.

### 2.5 Adenosine can be Produced by Astrocyte Activation Through ATP Hydrolysis and Exerts a Wake‐Promoting Role Through A_1_ Receptors

**Figure 5:**
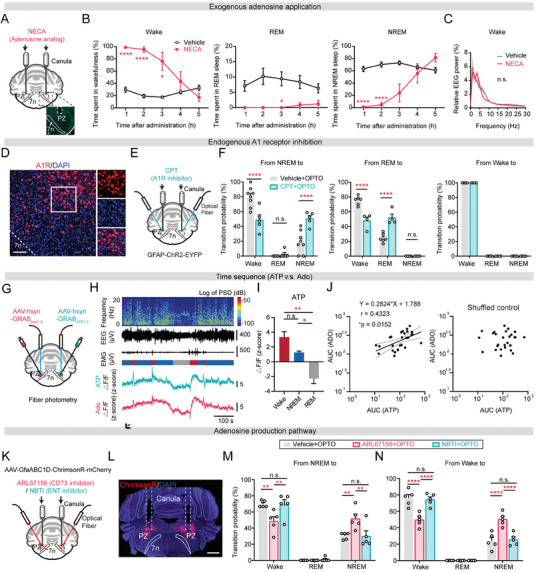
Adenosine generated through ATP hydrolysis exerts its wakefulness‐promoting effects by binding to A_1_ receptors. A) Setup for local drug delivery through bilateral canula on top of PZ in C57BL/6J mice (top). Representative image of canula location (bottom). Scale bar, 100 µm. B) Hourly percentage of time spent in wakefulness, REM sleep and NREM sleep in 5 h after the administration of NECA (*n =* 6 mice) or vehicle (*n =* 6 mice). Two‐way ANOVA test, Sidak's multiple comparisons test. Wake: ^*^ *p* = 0.0496, ^****^ *p* < 0.0001; REM: ^*^ *p* = 0.0433; NREM: ^****^ *p* < 0.0001. C) Relative EEG power (0‐30 Hz) during wakefulness after the administration of NECA (*n =* 4 mice) or vehicle (*n =* 4 mice). Two‐way ANOVA test, Sidak's multiple comparisons test. n.s., *p* = 0.4756. D) Representative image of A1 receptor expression in the PZ by immunohistochemistry staining of A_1_ receptors (red) and DAPI (blue) and a zoom‐in graph (right). Scale bar, 100 µm. E) Setup for optogenetic activation of astrocytes in the PZ following the administration of CPT (A_1_ receptor inhibitor). F) Probability of state transitions during optogenetic activation of PZ astrocytes following the administration of CPT or vehicle. From NREM to: vehicle group (*n =* 8 rats), CPT group (*n =* 5 rats); From REM or Wake to: vehicle group (*n =* 6 rats), CPT group (*n =* 4 rats). Two‐way ANOVA test, Sidak's multiple comparisons test, ^****^ *p* < 0.0001. G) Setup for simultaneous bilateral fiber photometry recordings of ATP and adenosine in the PZ throughout sleep‐wake cycles by recording GRAB_ATP1.0_ and GRAB_Ado1.0_ signals. H) Top to bottom, EEG power spectrogram (0–20 Hz), EEG traces, EMG traces, representative GRAB_ATP1.0_ (ATP) and GRAB_Ado1.0_ (Ado) fluorescence traces (z‐score) during sleep‐wake cycles; color code indicates NREM sleep, REM sleep, and wakefulness. I) Mean (±s.e.m.) ΔF/F (z‐score) of GRAB_ATP1.0_ (ATP) signals during each state. *n =* 3 mice. A one‐way ANOVA test, Tukey's multiple comparisons test, ^**^ *p* = 0.0080 (Wake vs REM), ^*^ *p* = 0.0390 (NREM vs REM). J) Correlation analysis of GRAB_ATP1.0_ and GRAB_Ado1.0_ signals (left) and the shuffled control (right). *n =* 31 trials, recorded from 3 individuals. Pearson correlation analysis, ^*^ *p* = 0.0152, r = 0.4323, red line shows linear regression line (Y = 0.2824*X+1.788), dot line indicates 95% regression range. K) Setup for optogenetic activation of astrocytes in the PZ following the administration of ARL67156 (CD73 inhibitor), NBTI (ENT inhibitor), or vehicle. L) Representative image of the expression of AAV‐GfaABC1D‐ChrimsonR‐mCherry in the PZ and the location of bilateral canula. Scale bar, 100 µm; red, ChrimsonR; blue, DAPI. M‐N) Probability of state transitions during optogenetic activation of PZ astrocytes following the administration of ARL67156 (*n =* 5 mice), NBTI (*n =* 5 mice), or vehicle (*n =* 5 mice). Two‐way ANOVA test, Tukey's multiple comparisons test. From NREM to Wake: ^**^ *p* = 0.0029 (Vehicle+OPTO vs ARL67156+OPTO), ^**^ *p* = 0.0044 (ARL67156+OPTO vs NBTI+OPTO); From NREM to NREM, ^**^ *p* = 0.0029 (Vehicle + OPTO vs ARL67156+OPTO), ^**^ *p* = 0.0026 (ARL67156+OPTO vs NBTI+OPTO). ^****^ *p* < 0.0001.

To examine the role of adenosine in the PZ in sleep‐wake regulation, we initially administered the adenosine analog 5'‐N‐ethylcarboxamidoadenosine (NECA) bilaterally through canula into the PZ of freely moving mice and rats to exogenously increase adenosine level (**Figure** **5**A). Strikingly, within 3 h following NECA injection, a substantial increase in wakefulness was observed at the expense of both NREM and REM sleep (Figure 5B; Figure S8A–C, Supporting Information). Additionally, the EEG power during wakefulness after NECA administration did not remarkably differ from natural wakefulness (Figure 5C). To gain further insights into the role of endogenous adenosine within the PZ, we first examined the distribution of adenosine A_1_ and A_2A_ receptors, known to be crucial in sleep regulation. Our immunostaining results for A_1_ receptors demonstrated a robust distribution within the PZ (Figure 5D). Conversely, using more sensitive RNAscope in situ hybridization approach, we observed no A_2A_ receptors expression in the PZ but dense expression in the caudate putamen (CPu), indicating an absence of A_2A_ receptor expression in the PZ (Figure S7A, B, Supporting Information). Moreover, activating A_1_ receptors by the A_1_‐specific agonist 2‐Chloro‐N6‐cyclopentyladenosine (CCPA) enhanced wakefulness, while inhibiting them with the A_1_‐specific antagonist 8‐cyclopentyl‐1,3‐dimethylxanthine (CPT) increased sleep (Figure S8G–I, Supporting Information). In contrast, the activation of A_2A_ receptors by injecting the A_2A_‐specific agonist CGS‐21680 showed no significant effect (Figure S8D–F, Supporting Information). Then, we examined whether endogenous adenosine acting on A_1_ receptors is responsible for the wake‐promoting role of astrocytes by optogenetically activating astrocytes after CPT administration through canula (Figure 5E). Notably, a significant reduction in the likelihood of transitioning from NREM or REM sleep to wakefulness was observed following A_1_ receptor inhibition (Figure 5F), implying the pivotal role of A_1_ receptors in astrocyte‐induced arousal.

Furthermore, we investigated the involvement of ATP in adenosine generation during astrocyte‐induced arousal. Initially, we compared the temporal dynamics between ATP and adenosine during sleep‐wake cycles. We injected GRAB_ATP1.0_ and GRAB_Ado1.0_ separately into each side of the PZ and simultaneously recorded their signals by fiber photometry (Figure 5G). Similar state‐dependent fluctuations were revealed for GRAB_ATP1.0_ and GRAB_Ado1.0_ (Figure 5H). We found a correlation between these 2 signals (r = 0.4323, Figure 5J left). Such correlation was not observed in the control analysis, in which the GRAB_Ado1.0_ signals were temporally shuffled in a random manner (r = 0.02912, Figure 5J right). To further explore the source of extracellular ATP, we simultaneously recorded PZ astrocytic Ca^2+^ activity and extracellular ATP levels during sleep‐wake cycles using fiber photometry. We injected AAV encoding GCaMP6f and the ATP sensor (AAV‐hsyn‐GRABATP1.0) into both sides of the PZ and implanted optic fibers above the region (Figure S9A). After a 3‐week viral expression period, we assessed changes in Ca^2+^ activity and extracellular ATP levels and found a correlation between these two signals (r = 0.4453, Figure S9B). Such correlation was not observed in the control analysis, in which the GRAB_ATP1.0_ signals were temporally shuffled in a random manner (r = ‐0.09599, Figure S9C).

Adenosine can be released into the extracellular space via two pathways: either hydrolyzed from extracellular ATP by ecto‐hydrolases (CD73/39) or transported by equilibrative nucleoside transporter (ENT).^[^ ^9^ ^]^ To ascertain which pathway contributes to adenosine production in the PZ, we injected ARL67156 trisodium salt hydrate (a CD73 inhibitor) or nitrobenzylthioinosine (NBTI, an ENT inhibitor) into the PZ via canula. Both compounds resulted in a noteworthy reduction in wakefulness, with a concurrent extension of time spent in NREM sleep (Figure S8J–L, Supporting Information). Furthermore, we tested which pathway is implicated in the wake‐promoting role of astrocytes by optogenetic activation of astrocytes after ARL67156 or NBTI administration. We bilaterally injected AAV encoding ChrimsonR (AAV2/5‐GfaABC1D‐ChrimsonR‐mCherry) into the PZ 3 weeks prior to drug administration (Figure 5K). The specific expression of ChrimsonR and the placement of canula were validated by post hoc immunostaining (Figure 5L; Figure S10B, Supporting Information). We also assessed the range of ChrimsonR expression in the PZ (Figure S2D, Supporting Information). Astrocytic Ca^2+^ activity was robustly increased following optogenetic activation by ChrimsonR (635 nm, 5 mW, 15 s, direct current) (Figure S10A–F, Supporting Information). Interestingly, compared to NBTI injection, the probabilities of transitioning from NREM sleep to wakefulness following ARL67156 injection were significantly reduced (Figure 5M,N). This suggests that adenosine produced via ATP hydrolysis is pivotal in the wake‐promoting effect of astrocyte activation.

In summary, these results indicate that astrocyte activation in the PZ primarily generates adenosine through ATP hydrolysis, and adenosine exerts a wake‐promoting role through A_1_ receptors in the PZ.

### 2.6 Adenosine Elevation in the PZ is Mainly Driven by Astrocyte Ca^2+^ Activity

**Figure 6:**
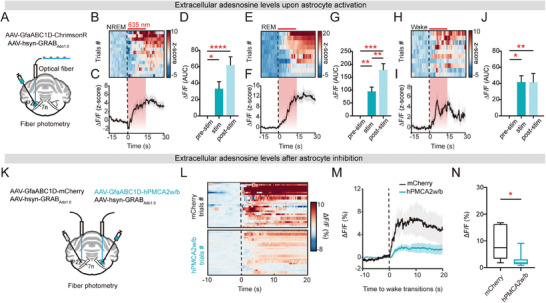
Astrocytes located in the PZ significantly contribute to the elevation of adenosine levels during wakefulness. A) Setup for fiber photometric recording of adenosine in the PZ while activating astrocytes optogenetically through a combined injection of GRAB_Ado1.0_ with ChrimsonR virus. B‐J) Heatmaps show GRAB_Ado1.0_ fluorescence traces during the 15 s before and 30 s after optogenetic stimulation in NREM sleep (B), REM sleep (E), and wakefulness (H). Line plots are mean ΔF/F (±s.e.m.) under optogenetic stimulation in NREM sleep (C), REM sleep (F), and wakefulness (I). Area under curve (AUC) of GRAB_Ado1.0_ signals in 15 s of pre‐, during, and post‐stimulation periods. D, NREM: *n =* 14 trials from 13 mice, Friedman test, Dunn's multiple comparisons test ^*^ *p* = 0.0245, ^****^ *p* < 0.0001; G, REM: *n =* 10 trials from 9 mice, a one‐way ANOVA test, Tukey's multiple comparisons test, ^**^ *p* = 0.0013 (pre‐stim vs stim), ^***^ *p* = 0.0002 (pre‐stim vs post‐stim), ^**^ *p* = 0.0012 (stim vs post‐stim); J, Wake: *n =* 10 trials from 10 mice, a one‐way ANOVA test, Tukey's multiple comparisons test, ^**^ *p* = 0.0018, ^*^ *p* = 0.0120. K) Setup for bilateral fiber photometry recording of GRAB_Ado1.0_ signals in the PZ, while inhibiting unilateral astrocytic Ca^2+^ activity through hPMCA2w/b expression and mCherry labeling contralaterally as control. L) Heatmaps show GRAB_Ado1.0_ fluorescence traces during the 10 s before and 20 s after transitions to wakefulness between hPMCA2w/b and mCherry group. M) Mean ΔF/F (±s.e.m.) during the 10 s before and 20 s after transitions to wakefulness of hPMCA2w/b (*n =* 10 mice) and mCherry group (*n =* 6 mice). N) Mean ΔF/F (±s.e.m.) of GRAB_Ado1.0_ signals between hPMCA2w/b (*n =* 10 mice) and mCherry group (*n =* 6 mice) during wakefulness. A two‐tailed Mann–Whitney test, ^*^ *p* = 0.0160.

We used an optogenetic fiber photometry approach to establish a causal relationship between astrocytic Ca^2+^ activity and adenosine with more precise temporal resolution. By mixed injection of ChrimsonR with GRAB_Ado1.0_, we measured real‐time extracellular adenosine changes upon optogenetic activation of astrocytes in the PZ (**Figure** **6**A). Notably, separate stimulations of astrocytes during NREM, REM sleep, and wakefulness consistently resulted in a notable surge in adenosine levels upon optogenetic activation (Figure 6B–J). It was verified that the laser stimulation itself did not significantly elevate adenosine levels (Figure S11, Supporting Information).

We next investigated whether astrocytic Ca^2+^ activity is a prerequisite for extracellular adenosine elevation. Employing hPMCA2w/b (AAV2/5‐GfaABC1D‐hPMCA2w/b‐mCherry) with GRAB_Ado1.0_ and mCherry (AAV2/5‐GfaABC1D‐mCherry) with GRAB_Ado1.0_ contralaterally as control in the PZ, we conducted real‐time bilateral adenosine fiber photometry recordings (Figure 6K). The inhibition of astrocytes by hPMCA2w/b led to a significant reduction in adenosine levels (Figure 6L–N). Taken together, these findings support a causal relationship between astrocytic Ca^2+^ activity and the extracellular adenosine, highlighting the critical and indispensable role of astrocyte activation in adenosine generation during sleep‐wake cycles.

### 2.7 Astrocyte Activation Inhibits the NREM‐Promoting and PB‐Projecting PZGABA Neurons

To investigate the specific neuronal types affected by astrocyte activation in the PZ, we examined the neuronal distribution of A_1_ receptors in the PZ, revealing that 70.56% of A1R‐positive neurons are GABAergic, while 29.44% are glutamatergic (Figure S7C–F). Furthermore, previous studies have reported that chemogenetic activation of PZ^Phox2B^ neurons does not affect the sleep‐wake phenotype (Phox2B is a location‐specific marker for PZ^Vglut2^ neurons),^[^ ^21^ ^]^ suggesting that PZ glutamatergic neurons are insufficient to influence the sleep‐wake cycle. Thus, we hypothesize that astrocytes may primarily affect the sleep‐wake phenotype by regulating the activity of PZ GABAergic neurons.

**Figure 7:**
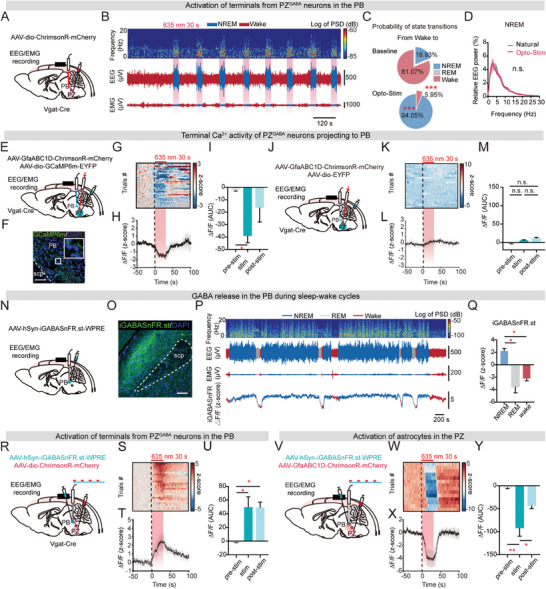
Astrocyte activation inhibits NREM‐promoting and PB‐projecting GABAergic neurons in the PZ. A) Setup for optogenetic activation of terminals from PZ^GABA^ neurons projecting to the PB in combination with EEG/EMG recordings during sleep‐wake cycles. B) Top to bottom, EEG power spectrogram (0–20 Hz), EEG traces, and EMG traces throughout sleep‐wake cycles; color code indicates NREM sleep and wakefulness. Red shade indicates optogenetic activation (635 nm, 1 mW, 30 s, 40 Hz, 5 ms). C) Comparison of the probability of state transitions between the baseline condition and optogenetic activation. Two‐way ANOVA test, Sidak's multiple comparisons test, ^***^ *p* = 0.0002. D) Relative EEG power (0–30 Hz) during natural NREM sleep (*n =* 5 mice) and optogenetic activation (*n =* 5 mice). Two‐way ANOVA test, Bonferroni's multiple comparisons test. E) Setup for fiber photometric recording of the terminal Ca^2+^ activity from PZ^GABA^ neurons projecting to the PB in response to the optogenetic activation of PZ astrocytes. F) Representative image of the terminal CCaMP6 m expression from PZ^GABA^ neurons projecting to the PB (position of coronal section). Scale bar, 50 µm; green, CCaMP6 m; blue, DAPI. G) Heatmap shows CCaMP6 m fluorescence traces during the 50 s before and 100 s after optogenetic activation of PZ astrocytes. *n =* 3 mice. H) Mean (±s.e.m.) ΔF/F (z‐score) during the 50 s before and 100 s after optogenetic activation (red shade) of PZ astrocytes. *n =* 3 mice. I) Area under curve (AUC) of CCaMP6 m signals in 30 s of pre‐, during, and post‐stimulation periods. *n =* 3 mice, a one‐way ANOVA test, Tukey's multiple comparisons test, *p = 0.0326 (pre‐stim vs stim). J) Setup for fiber photometric recording of the terminal EYFP fluorescence from PZ^GABA^ neurons projecting to the PB in response to the optogenetic activation of PZ astrocytes. K) Heatmap shows EYFP fluorescence traces during the 50 s before and 100 s after optogenetic activation of PZ astrocytes. *n =* 3 mice. L) Mean (±s.e.m.) ΔF/F (z‐score) during the 50 s before and 100 s after optogenetic activation (red shade) of PZ astrocytes. *n =* 3 mice. M) AUC of EYFP fluorescence in 30 s of pre‐, during, and post‐stimulation periods. *n =* 3 mice, a one‐way ANOVA test, Tukey's multiple comparisons test. N) Setup for fiber photometric recording of extracellular GABA in the PB by recording iGABASnFR.st signals in combination with EEG/EMG recordings during sleep‐wake cycles. O) Representative image of iGABASnFR.st expression in the PB (position of coronal section). Scale bar, 100 µm; green, iGABASnFR.st; blue, DAPI. P) Top to bottom, EEG power spectrogram (0‐20 Hz), EEG traces, EMG traces, and representative iGABASnFR.st fluorescence traces (z‐score) during sleep‐wake cycles; color code indicates vigilant states. Q) Mean ΔF/F (z‐score) of iGABASnFR.st signals during NREM sleep, REM sleep, and wakefulness. *n =* 3 mice, a one‐way ANOVA test, Tukey's multiple comparisons test, ^*^ *p* = 0.0357 (NREM vs Wake), ^*^ *p* = 0.0147 (NREM vs REM). R) Setup for fiber photometric recording of extracellular GABA in the PB in response to the optogenetic activation of terminals from PZ^GABA^ neurons projecting to the PB. S) Heatmap shows iGABASnFR.st fluorescence traces during the 50 s before and 100 s after optogenetic activation of terminals from PZ^GABA^ neurons projecting to the PB. *n =* 3 mice. T) Mean (±s.e.m.) ΔF/F (z‐score) during the 50 s before and 100 s after optogenetic activation (red shade) of terminals from PZ^GABA^ neurons projecting to the PB. *n =* 3 mice. U) AUC of iGABASnFR.st signals in 30 s of pre‐, during and post‐stimulation periods. *n =* 3 mice, a one‐way ANOVA test, Tukey's multiple comparisons test, ^*^ *p* = 0.0193 (pre‐stim vs stim), ^*^ *p* = 0.0201 (pre‐stim vs post‐stim). V) Setup for fiber photometric recording of extracellular GABA in the PB in response to the optogenetic activation of astrocytes in the PZ. W) Heatmap shows iGABASnFR.st fluorescence traces during the 50 s before and 100 s after optogenetic activation of astrocytes in the PZ. *n =* 3 mice. X) Mean (±s.e.m.) ΔF/F (z‐score) during the 50 s before and 100 s after optogenetic activation (red shade) of astrocytes in the PZ. *n =* 3 mice. Y) AUC of iGABASnFR.st signals in 30 s of pre‐, during and post‐stimulation periods. *n =* 4 mice, a one‐way ANOVA test, Tukey's multiple comparisons test, ^**^ *p* = 0.0021 (pre‐stim vs stim), ^*^ *p* = 0.0340 (stim vs post‐stim).

Prior electrophysiological studies have presented compelling evidence suggesting that GABAergic neurons within the PZ promote NREM sleep, most likely by inhibiting the wake‐promoting glutamatergic neurons in the PB.^[^ ^19^ ^]^ To explore the neural circuitry influenced by PZ astrocytes in sleep‐wake cycles, our primary focus was on validating the hypothesized role of PZ^GABA^ neurons projecting to the PB in regulating sleep‐wake behavior. AAV encoding Cre‐dependent ChrimsonR (AAV2/9‐dio‐ChrimsonR‐mCherry) was injected into the PZ of Vgat‐IRES‐Cre mice and the terminals of PZ^GABA^ neurons projecting to the PB were optogenetically activated (635 nm, 1 mW, 30 s, 40 Hz, 5 ms) (**Figure** **7**A). Our results demonstrated that optogenetic activation of terminals of PZ^GABA^ neurons projecting to the PB steadily elicited NREM sleep (Figure 7B,C), and the EEG patterns during induced NREM sleep closely resembled those of natural NREM sleep (Figure 7D).

To investigate the impact of astrocyte activation on this group of PB‐projecting, NREM sleep‐promoting PZ^GABA^ neurons, we measured terminal Ca^2+^ activity from PZ^GABA^ neurons projecting to the PB in response to optogenetic activation of PZ astrocytes. We injected AAV encoding Cre‐dependent GCaMP6 m (AAV2/9‐dio‐GCaMP6 m) into the PZ of Vgat‐IRES‐Cre mice and monitored Ca^2+^ activity across sleep‐wake cycles using fiber photometry (Figure 7E). We validated terminal GCaMP6 m expression in the PB by post hoc immunostaining (Figure 7F). Interestingly, we found that Ca^2+^ activity significantly decreased upon optogenetic activation of astrocytes (Figure 7G–I). To confirm this effect, we also recorded terminal EYFP fluorescence upon optogenetic activation of PZ astrocytes in control group, which did not induce a fluorescence reduction in the PB (Figure 7J–M).

We further investigated the impact of PZ astrocyte activation on extracellular GABA release in the PB. iGABASnFR is an intensity‐based GABA‐sensing fluorescence reporter and the fluorescence change of iGABASnFR was restricted to the membrane.^[^ ^29^ ^]^ To continuously monitor real‐time GABA levels in the extracellular space during sleep‐wake cycles, we injected AAV encoding GABA sensor (AAV2/9‐hSyn‐iGABASnFR.st‐WPRE‐pA) into the PB (Figure 7N). Post hoc immunostaining confirmed the robust expression of iGABASnFR.st within the PB (Figure 7O). Notably, in natural sleep‐wake cycles, GABA levels remained stable during NREM sleep but significantly decreased during REM sleep and wakefulness (Figure 7P,Q). To validate the reliability of iGABASnFR.st signals, we injected AAV expressing Cre‐dependent ChrimsonR (AAV2/9‐dio‐ChrimsonR‐mCherry) into the PZ and the iGABASnFR.st into the PB of Vgat‐IRES‐Cre mice, monitoring GABA using an optical fiber positioned above the PB (Figure 7R). We found that the optogenetic activation of PB‐projecting terminals from PZ^GABA^ neurons (635 nm, 1 mW, 30 s, 40 Hz, 5 ms) led to a substantial increase in extracellular GABA levels in the PB (Figure 7S–U). Subsequently, we respectively injected the iGABASnFR.st into the PB and AAV2/5‐GfaABC1D‐ChrimsonR‐mCherry into the PZ and optimized with a thick optical fiber above the PB (diameter: 400 µm, covering both the PB and PZ in the rostral‐caudal direction) (Figure 7V). This setup allowed real‐time recording of GABA in the PB following the optogenetic activation of PZ astrocytes. Notably, GABA levels in the PB exhibited a significant decrease upon optogenetic activation of PZ astrocytes (Figure 7W–Y).

In summary, the remarkable decline of GABA release in the PB following PZ astrocyte activation highlights a pivotal inhibitory role of PZ astrocytes on the PB‐projecting PZ^GABA^ neurons which are proven to be NREM sleep‐promoting in the present study.

## 3 Discussion

In the present study, astrocyte activation within the PZ evokes and sustains arousal at the cost of both NREM and REM sleep. Intriguingly, adenosine plays a role in promoting wakefulness in the PZ through A_1_ receptors, suggesting a distinct role for adenosine in the PZ beyond its conventional involvement in sleep homeostasis modulation observed in the BF and cortex. Furthermore, this extracellular adenosine originates from astrocytes, and astrocyte activation specially triggers CD73/39‐mediated adenosine production, contributing to arousal promotion. Astrocyte activation suppresses the GABA release in the PB from the PZ^GABA^ neurons, thus shedding light on the involvement of astrocyte‐neuron communication in maintaining arousal at the circuit level (Figure S12, Supporting Information).

### 3.1 Arousal Induced by Astrocyte Activity in Sleep‐Wake Cycle

Astrocytes have emerged as pivotal regulators of sleep through distinct pathways, encompassing the modulation of sleep homeostasis,^[^ ^30^ ^]^ waste removal via the glymphatic system,^[^ ^31^ ^]^ and the provision of lactate to neurons.^[^ ^32^ ^]^ Notably, a substantial increase in astrocytic Ca^2+^ activity and gliotransmission during wakefulness, in contrast to NREM sleep, has been consistently observed throughout the brain, underlining the equally critical role of astrocytes in the regulation of wakefulness.^[^ ^11^, ^12^, ^33^ ^]^ Our data clearly demonstrate the indispensability of astrocytes in initiating and sustaining wakefulness (Figures 2 and 3). Interestingly, the inhibition of astrocytes primarily affected the first 1–2 h after the transition into the AP in which the wakefulness duration and level are relatively high, resulting in increased sleep in the mice. This suggested that PZ astrocytic Ca^2+^ activity may be critical to the maintenance of high‐level wakefulness.

### 3.2 Spotlighting the Wake‐Promoting Effects of Adenosine

Adenosine has traditionally been regarded as a sleep‐promoting factor in the context of sleep pressure accumulation.^[^ ^30^, ^34^ ^]^ However, our research, supported by a growing body of literature, suggests that this traditional perspective may not entirely hold true.^[^ ^35^, ^36^, ^37^ ^]^ The impact of adenosine on sleep appears to exhibit brain region‐dependent variations and engage distinct mechanisms.^[^ ^38^, ^39^ ^]^ Elevated extracellular adenosine levels during prolonged wakefulness, observed in the BF and cortex, do not occur uniformly throughout the brain.^[^ ^35^, ^39^ ^]^ This observation suggests that in brain regions other than the BF and cortex, adenosine might exert effects beyond sleep homeostasis modulation.

The adenosine‐mediated effects on sleep‐wake cycles are receptor‐dependent. Adenosine A_1_ and A_2A_ receptors are believed to regulate sleep via dissociable effects, that A_2A_ receptors drive the brain to sleep while A_1_ receptors modulate sleep.^[^ ^40^, ^41^ ^]^ Additionally, A_1_ and A_2A_ receptors are differentially distributed and expressed across the brain.^[^ ^37^ ^]^ Systemic administration of caffeine induces arousal primarily by antagonizing A_2A_ receptors instead of A_1_ receptors.^[^ ^42^ ^]^ While A_1_ receptors are essential for sleep homeostasis regulation, their activation in the brainstem has been observed to promote arousal in rats^[^ ^43^ ^]^ and cats.^[^ ^44^ ^]^ Our study unveils a novel role of adenosine in the PZ: it induces arousal through activating A_1_ receptors, distinct from the somnogenic effect observed in other brain regions such as the BF,^[^ ^45^ ^]^ tuberomamillary nucleus (TMN),^[^ ^46^ ^]^ and LH.^[^ ^47^ ^]^ The activation of A_1_ receptors is believed to inhibit adenylate cyclase, leading to a reduction in the second messenger cyclic adenosine monophosphate (cAMP), and this, in turn, inhibits voltage‐gated Ca^2+^ channels and activates G‐protein‐coupled inwardly rectifying K^+^ channels through Gi/o βγ‐subunits, resulting in decreased neuronal excitability.^[^ ^23^ ^]^ Moreover, the availability and activation levels of A_1_ receptors increase in response to persistent wakefulness in both mice^[^ ^33^ ^]^ and humans.^[^ ^48^ ^]^ This implies that when contemplating the utilization of adenosine for addressing sleep disorders or treating emotionally related conditions such as anxiety disorders,^[^ ^49^ ^]^ this factor should be duly considered.

### 3.3 Unveiling Astrocytic Regional Variations in Sleep‐Wake Regulation

In our work, astrocytic Ca^2+^ activity has been identified to fluctuate in tandem with state transitions, thereby modulating sleep behavior. Importantly, this correlation varies across different brain regions.^[^ ^12^ ^]^ Astrocytic Ca^2+^ activities exhibited variable patterns in the cortex, hippocampus, hypothalamus, pons and cerebellum throughout the sleep‐wake cycle.^[^ ^10^, ^11^, ^12^ ^]^ In the PZ, we observed that astrocytic Ca^2+^ activity is highest during wakefulness and lowest during REM sleep, mirroring the astrocytic Ca^2+^ dynamics observed in the brainstem.^[^ ^12^ ^]^ Furthermore, our data support the notion that astrocyte activation yields distinct sleep‐regulating effects in different brain regions, promoting wakefulness in the PZ and LH regions while having no significant impact in the BF (Figure 2; Figure S5, Supporting Information). On the contrary, it has been reported that chronic optogenetic activation of astrocytes in the VLPO and posterior thalamus increases NREM sleep.^[^ ^15^, ^16^ ^]^ In our study, optogenetic activation of LH astrocytes via ChrimsonR resulted in arousal but not NREM sleep. Previous researches show that endogenous adenosine in the LH, acting through A1 receptors, could act on orexinergic,^[^ ^50^ ^]^ glutamatergic,^[^ ^51^ ^]^ and GABAergic^[^ ^52^ ^]^ neurons to regulate sleep. Therefore, further research is needed to determine the specific mechanism by which LH astrocytes promote arousal. In the BF, glutamatergic neurons, rather than cholinergic (Ach) neurons, have been shown to regulate adenosine production.^[^ ^53^ ^]^ Moreover, astrocytes in the BF may not be the primary source of adenosine, as their activation does not significantly increase adenosine levels.^[^ ^54^ ^]^ This implies that astrocytes are not the sole source of adenosine and play different roles in sleep in different brain regions. In the brain, both adenosine and ATP can originate from various cell types, including astrocytes, neurons, and endothelial cells. These region‐dependent effects align with our findings, underscoring the complexity and diversity of astrocytic roles in sleep regulation.

Several lines of evidence substantiate this observation. Initially, the abundance of differentially expressed genes in astrocytes across distinct brain regions, signifies the existence of region‐specific microenvironments and functions. This differential gene expression includes those related to Ca^2+^ flux pathways and G‐protein‐coupled receptor (GPCR) signaling, which regulates intracellular Ca^2+^ activity in astrocytes.^[^ ^3^, ^55^, ^56^ ^]^ Furthermore, even within the same brain region, distinct astrocyte subclusters have been identified, each proportionally represented in diverse brain regions. Enrichment of subcluster‐specific genes related to neurotransmitter homeostasis, synapse function, and phagocytosis has been documented, indicating a region‐dependent astrocyte‐neuron communication.^[^ ^55^, ^57^ ^]^ Considering the heterogeneity of astrocytes in cellular, molecular and functional properties across the entire brain,^[^ ^3^, ^55^, ^56^, ^57^, ^58^ ^]^ it is conceivable that these cells undertake distinct physiological roles in regulating the sleep‐wake cycle.

### 3.4 Astrocytic Modulation of Synaptic Homeostasis During Sleep‐Wake Cycle at the Brainstem Neuronal Circuit

Astrocytes has been discovered to stabilize, potentiate, or even shape neuronal circuits across a range of behaviors throughout the nervous system.^[^ ^2^, ^3^, ^59^ ^]^ Our data reveals that astrocytes regulate inhibitory synaptic transmission by releasing adenosine via A_1_ receptors, thereby inhibiting the activity of PZ^−PB^ GABAergic neurons and promoting wakefulness. A_1_ receptors are believed to exert inhibitory effects primarily on presynaptic neurons, leading to the downregulation of neurotransmitter release.^[^ ^60^ ^]^ This reaffirms the pivotal role of astrocyte‐neuron crosstalk in maintaining synaptic homeostasis. Our study represents a pioneering discovery, demonstrating that astrocytes possess the capacity to regulate behavior at the level of a neural circuit.

We acknowledge several limitations in our current study. First, we did not fully explore the role of adenosine in the PZ‐PB circuit. We have designed experiments to knockout the A1R in PZ GABAergic neurons and observe the effect on astrocytic‐activation‐induced wakefulness. Unfortunately, the GABAergic neuron‐specific A1 knockout strategy is still unsatisfactory owing to the low specificity. Additionally, our experiments were solely conducted on male rodents because the sleep behavior of female animals is easily influenced by the menstrual cycle and sex hormones, which limits the broader applicability of our findings.

## 4 Conclusion

In summary, our study demonstrates a distinctive arousal‐promoting effect of astrocytes within the PZ via extracellular adenosine through A_1_ receptors at the circuit level, which extends beyond the traditional role of adenosine in modulating sleep homeostasis in the BF and cortex. This indicates that astrocytes may exert region‐specific influences on sleep and wakefulness via distinct mechanisms.

## 5 Experimental Section

### Animals

All experimental procedures were conducted in accordance with the Guidelines for the Care and Use of Laboratory Animals and was approved by Zhejiang University (ZJU20230509). Adult C57BL/6J, Vgat‐IRES‐Cre (Jackson, strain name *Slc32a1^2(cre)Lowl^ /J*, stock number 016962) mice were used for the experiments. C57BL/6J mice were purchased from Vital River Experiment Animal Co., Ltd. 6‐ to 16‐week‐old male C57BL/6J mice of similar body weight were used depending on the specific experiment. For in vivo optogenetic astrocyte stimulations, astrocyte‐specific ChR2‐expressing rats (GFAP‐ChR2‐EYFP rats) were used with Sprague–Dawley background generated by the Institute of Neuroscience, Chinese Academy of Sciences.^[^ ^22^ ^]^ A single guide (sg) RNA near the stop codon in the last exon of the GFAP gene was designed and constructed a donor plasmid containing the ChR2‐EYFP sequence, which was used as a template to repair the double‐strand break by homologous recombination. Super‐ovulated female Sprague–Dawley rats were mated to Sprague–Dawley males, and fertilized embryos were collected and Cas9 mRNA, sgRNAs, and donor were mixed and injected into the cytoplasm of fertilized eggs. Afterward, 20–25 embryos were transferred into the oviducts of pseudo‐pregnant Sprague–Dawley rats. All experiments were conducted on 2‐ to 4‐month‐old male and female GFAP‐ChR2‐EYFP rats and WT Sprague‐Dawley rats. Animals were housed and maintained in a temperature and humidity controlled environment at 22 ± 1 °C and 55 ± 5% humidity with 12 h light/dark cycles (lights on at 07:00, ZT0). They were fed regular rodent chow and tap water ad libitum.

### Viral Delivery and Stereotaxic Surgeries

AAV‐GfaABC1D‐lck‐GCaMP6f‐SV40‐EGFP (AAV2/5, 2.0 × 10^12^ genomic copies mL^−1^), AAV‐GfaABC1D‐hM3Dq‐mCherry (AAV2/5, 5.90 × 10^12^ genomic copies mL^−1^), were provided by BrainVTA (Wuhan) Co., Ltd. (Wuhan, China). AAV2/9‐hEF1α‐DIO‐hChR2 (H134R)‐EYFP (AAV2/9, 1.96 × 10^13^ genomic copies mL^−1^), AAV‐hSyn‐DIO‐GCaMP6m‐WPRE (AAV2/9, 1.04 × 10^13^ genomic copies mL^−1^), and AAV‐hSyn‐iGABASnFR.st‐WPRE‐pA (AAV2/9, 1 × 10^13^ genomic copies mL^−1^), AAV‐hSyn‐DIO‐ChrimsonR‐mCherry (AAV2/9, 2 × 10^13^ genomic copies mL^−1^), and AAV2/9‐hSyn‐DIO‐mCherry (AAV2/9, 1.48 × 10^13^ genomic copies mL^−1^) were made by Shanghai Taitool Bioscience Co., Ltd. (Shanghai, China). pZac2.1‐GfaABC1D‐mCherry‐hPMCA2w/b was from Baljit Khakh Lab Plasmids in Addgene (Addgene plasmid # 111568).^[^ ^28^ ^]^ AAV‐GfaABC1D‐hPMCA2w/b‐mCherry (AAV2/5, 7.0 × 10^12^ genomic copies mL^−1^) and AAV‐GfaABC1D‐mCherry (AAV2/5, 6.0 × 10^12^ genomic copies mL^−1^), AAV‐hsyn‐GRAB_Ado1.0_ (AAV2/9, 1.52 × 10^13^ genomic copies mL^−1^), AAV‐hsyn‐GRAB_Ado1.0mut_ (AAV2/9, 1.52 × 10^13^ genomic copies mL^−1^), AAV‐hsyn‐GRAB_ATP1.0_ (AAV2/9, 2 × 10^13^ genomic copies mL^−1^), AAV‐GfaABC1D‐ChrimsonR‐mCherry (AAV2/5, 1.51 × 10^13^ genomic copies mL^−1^) were constructed by Vigene Biosciences (Shandong, China).

Animals were anesthetized with 1% sodium pentobarbital and secured in a stereotaxic frame. Body temperature was kept stable throughout the surgery with a heating pad. 0.15–0.25 µL of the viral solution (depending on viral titer) was injected into each location at 0.01 mL min^−1^, and the microsyringe was kept still in 10 min after administration to minimize the upward flow of viral solution when removing the needle. The coordinates of virus injection locations in mice included PZ (AP, −5.50 mm; ML, ±1.4 mm; DV, −4.5 mm), PB (AP, −5.20 mm; ML, ±1.4 mm; DV, −3.5 mm), BF (AP, 0.02 mm; ML, ±1.45 mm; DV, −4.90 mm) and LH (AP, −1.10 mm; ML, ±1.2 mm; DV, −4.9 mm). The coordinate of PZ in rats was AP, −10.10 mm; ML, ±2.2 mm; DV, −8.1 mm. Coordinates of implanted canula or optical fiber were located 200 µm above the injection sites. After injection, 3 weeks recovery of animals were allowed before performing behavioral tests. Virus expression in the target region was confirmed by post hoc immunostaining, and mice showing no detectable viral expression in the target region were excluded.

### Surgical Procedures

For sleep recording experiments, four skull screw‐holes (two above the visual cortex and the other two above the frontal cortex) were drilled and four stainless steel screws were tightly driven through the skull to the surface of the dura. A custom‐made EEG and EMG recording unit was installed on top of the skull. Two stainless‐steel wires were placed into neck muscles as EMG electrodes. The whole implant was fixed to the skull with dental cement. After surgery, animals were allowed to recover in their individual chambers for at least 7 days. Thereafter, each animal was transferred to a new recording chamber connecting to an EEG/EMG head‐stage. A slip‐ring (CFS‐22) was connected to the cable on top of head‐stage to make sure that animals could move freely without tangling the cable. The animals were habituated in the recording chamber for 3 days before EEG and EMG recording.

For fiber photometry recording or optogenetic manipulation experiments, an optical fiber canula (core diameter 200 µm, NA 0.37; inper, China) was implanted 200 µm above the virus injection site unilaterally or bilaterally after viral injection. The optic fiber was first bonded to the skull by an adhesive (3M Vetbond) applied on the edges and then secured to the skull with dental cement. The implanted optical fiber was connected to a laser source by an optic fiber patch cord (inper, China). The light intensity was measured at the tip of the optical fiber by a laser power meter (LP1, Sanwa, Japan) before each test. After implantation, animals were allowed to recover for 7 days before behavioral tests. The positions of the optical fibers were confirmed by post hoc immunostaining. Animals with incorrect positions of optical fibers were excluded.

For drug administration experiments, double guide canula (I.D. 340 µm, RWD Life Science, China) were implanted at 200 µm above the coordinates of bilateral PZ. For drug administration coupled with optogenetics experiments, double guide canula (I.D. 450 µm) were implanted after optogenetic virus injection. The optic fiber patch cord was custom‐made to be able to insert into the double guide canula, which enables simultaneous optogenetic stimulation and drug infusion. The canula was fixed to the skull with dental cement. After implantation, animals were allowed to recover for 7 days before behavioral tests. The positions of canula were verified by post hoc immunostaining. Animals with incorrect positions of canula were excluded.

### EEG and EMG Recording

EEG and EMG signals from the implanted electrodes were amplified, filtered (EEG, band pass 0.5–50 Hz; EMG, high pass, > 10 Hz), digitized at 200 Hz, and recorded using Labchart (AD instrument, USA).

### Vigilant State Classification

Vigilant states were scored using professional sleep analysis software (SleepSign, Kissei Comtec). All scoring was automatically performed based on the EEG and EMG waveforms in 4 s epoch divisions. Vigilant state classification was performed according to an established criterion: Wakefulness was defined as desynchronized EEG and high tonic EMG activity with phasic bursts; NREM was defined as synchronized EEG with high‐amplitude delta activity (0.5–4 Hz) and remarkably reduced EMG activity compared with wakefulness with no phasic bursts; REM was defined as a pronounced theta rhythm (4–10 Hz) and a flat EMG (atonia). All classifications of states assigned by SleepSign were validated manually by trained experimenters. Detailed analyses of sleep structure and quality were also accomplished by SleepSign.

### EEG Power Spectral Analysis

The digitally‐filtered EEG signals were also spectrally analyzed with fast Fourier transformation (FFT) algorithm by SleepSign program. The EEG power spectral density analyses were accomplished with NeuroExplorer (Nex Technology). The slow wave power (delta wave power) was summed from the spectrum of 0.5–4 Hz in an overall 0.5–50 Hz window with 0.38 Hz resolution. The slow wave activity (%) was calculated by the slow wave power divided by the mean value of total power in the same time course. The relative EEG power (%) was used to normalize the data, represented by the ratio of the power of a specific frequency range in an epoch divided by the mean value of total power in the same epoch. To analyze the change of EEG power spectrum between induced and natural states, the relative EEG power of induced states (e.g., 15 s photostimulation) and natural states (e.g., 15 s pre‐photostimulation) was compared.

### Fiber Photometry Recording During the Sleep‐Wake Cycles

To record signal change of GCaMP, adenosine or ATP sensors during sleep‐wake cycles, an optical fiber (O.D. 200 µm, NA 0.37, inper) placed in a ceramic ferrule was implanted and the emitted fluorescence was recorded by fiber photometry. A tricolor multi‐channel fiber photometry system (Thinker Tech Nanjing Biotech Ltd, Nanjing, China) was used for recording. The tricolor multi‐channel fiber recording system adds 405 and 580 nm excitation light channels on the basis of the previous 470 nm excitation light, which can simultaneously record the green and red fluorescence of calcium signals and the signal of reference channel through the same fiber. The reference channel uses 405 nm excitation light, and the signal of the channel can be used as control data to eliminate movement noise and verify the validity of the data of the calcium signal channel. The system adopts CMOS imaging method to acquire the fluorescence brightness of each fiber in the multi‐mode fiber bundle in real‐time, so as to realize the synchronous recording of multiple channels. The fluorescence signals with intensity change is returned to the detection light path through the same fiber and collected by CMOS in the form of images, and extracted by acquisition software to display calcium signals.

During recording, the calcium signals were sampled at 40 Hz for each wavelength fluorescence and the digital signals were sampled at 120 Hz. To minimize GCaMP bleaching, the laser power at the tip of the optical fiber was adjusted to a low level (0.01–0.02 mW). To minimize the auto‐fluorescence of the optical fiber and cell debris, the recording fiber patch cord was bleached for 1 day before recordings and the first half‐hour data was not included in the subsequent analysis because of the autofluorescence of cell debris.

### Fiber Photometry Recording Coupled with Optogenetic Activation

To record optogenetic activation‐evoked GCAMP or other sensor signals change, a 593.5 nm laser was coupled into the optical path in the tricolor multi‐channel fiber photometry system, which can record GCaMP or sensor signals (405 nm and 470 nm light source is turned on for fiber recording) as well as connect to the laser for ChrimsonR activation simultaneously at the same brain region. To eliminate the laser‐induced artifact in the photometry signal, the change of EYFP fluorescence upon introducing the laser with the same parameters into the optical path was also recorded, and no significant change in fluorescence was detected. The timing of laser on for optogenetic stimulation was controlled by the Labchart, the EEG/EMG recording software which simultaneously monitored the vigilant states.

### Fiber Photometry Data Analysis

To analyze the data of fiber photometry, the fluorescence value of the reference 405 nm channel was first subtracted to minimize the artifacts of background autofluorescence and movement. The fluorescence change values (ΔF/F) were calculated as (V_signal_–F_0_)/(F_0_–V_offset_), in which F_0_ is the stable baseline fluorescence signal averaged over a 2 s/4 s time‐window prior to a trigger event and V_offset_ is the fluorescence signal recorded in the dark before connecting to the optical fiber. To minimize the variation of basal fluorescence in different animals and trials, normalized fluorescence change values (z‐score) were calculated as (V_signal_–F_0_)/σF, where σF is the standard deviation of the baseline fluorescence signal. Considering that signals at the beginning of each recording (−30 min) were often prone to a sharp decline, probably owing to the photobleaching of autofluorescence of the cell debris, data during the first half hour were thus excluded in the quantification.

To remove the slow drift in the photometry signal during long‐time fiber photometry recording, an algorithm named adaptive iteratively reweighted Penalized Least Squares (airPLS) that does not require any user intervention and prior information was used.^[^ ^61^ ^]^ It works by iteratively changing weights of sum squares errors (SSE) between fitted baseline and original signals, and the weights of SSE are obtained adaptively using the difference between previously fitted baseline and original signals.

To remove the recording noise and improve the signal‐to‐noise ratio, the wavelet denoising function in MATLAB was used to reveal the fluctuation of signals during sleep‐wake cycles.

To analyze the correlation of the GRAB_Ado1.0_ and GCaMP or GRAB_ATP1.0_ signals, the area under the curve of signals (AUC) was used to analyze the correlation between 2 signals. To improve clarity, a logarithmic transformation (base 10) was applied when plotting the correlation between GRAB_Ado1.0_ and GCaMP or GRAB_ATP1.0_ signals. The Pearson's r and the regression equation for the two signals were calculated to generate the scatter plots in Figures 4H and 5J (left) and Figure S9B (Supporting Information). To confirm the absence of non‐specific correlations, one signal was randomly shuffled, and the AUC was recalculated using the same method as the non‐shuffled condition, producing the scatter plots in Figures 4I and 5J (right) and Figure S9C (Supporting Information). The Pearson's r for the two signals was calculated using the raw AUC data (without log transformation).

To estimate time sequence of the GRAB_Ado1.0_ and GCaMP or GRAB_ATP1.0_ signals, the onset of GRAB_Ado1.0_ events were first defined as the time when the signal reaches 10% of its peak in the rising edge. The onset of GRAB_Ado1.0_ events were used as a reference to align the GCaMP or GRAB_ATP1.0_ with GRAB_Ado1.0_ signals. The time difference was estimated by the time difference when the signals climbed half of its peak.

### Pharmacogenetic Activation

To evaluate roles of astrocytes in sleep regulation by chronic activation, AAV‐GfaABC1D‐hM3Dq‐mCherry virus (200 nl per side) was injected bilaterally in the PZ. The control group were bilaterally injected with AAV‐GfaABC1D‐mCherry virus (200 nl per side) in the PZ. Three weeks after virus injection, mice were injected intraperitoneally with 0.2 mL saline or CNO (1 mg kg^−1^, Sigma, C0832, stored at −20 °C and dissolved in 0.9% sterile saline to a concentration of 1 mg mL^−1^ before use) at ZT6, sleep recording was performed during ZT0‐24 and each mouse has adapted to experimenter handling and the environment in the recording chamber for a week before recording.

To verify whether astrocytes were activated by hM3Dq after CNO injection, the Ca^2+^ activity of astrocytes was measured by mixed injection of AAV‐GfaABC1D‐hM3Dq‐mCherry (100 nl/side) and AAV‐GfaABC1D‐lck‐GCaMP6f‐SV40‐EGFP (100 nl/side) into PZ and an optical fiber was implanted on top of PZ. Three weeks after virus injection, mice were injected intraperitoneally with 0.2 mL saline or CNO (1 mg kg^−1^) at ZT6. The recording fiber patch cord was connected to the optical fiber immediately after injection.

### Optogenetic Activation

To acutely activate astrocytes in the PZ, GFAP‐ChR2‐EYFP rats was used and an optical fiber (core diameter 200 µm, NA 0.37; inper, China) was implanted unilaterally on top of PZ. Wild‐type SD rats implanted with an optical fiber unilaterally on top of PZ is regarded as the control group. After surgery, mice were allowed 1 week to recover. The optical fiber was connected to a 465 nm blue laser source (inper, China) using an optical fiber sleeve. The light intensity was the same as that optimized for activating astrocytes on previous testing days (5 mW, 15 s, direct current). Each rat was stimulated during different vigilant states for at least 8 trials per state in inactive period (IP, ZT0‐12) and active period (AP, ZT12‐24). Each stimulation was performed with a time interval for more than 3 min to ensure a full recovery for astrocytes. Rats with missed optical fiber placements were excluded. In the control group, SD rats underwent the same procedure and received the same intensity of laser stimulation.

To activate astrocytes more locally in the PZ, AAV‐GfaABC1D‐ChrimsonR‐mCherry (200 nl/side) bilaterally was injected into PZ in C57BL/6J mice and implanted optical fibers on top. In the control group, AAV‐GfaABC1D‐mCherry (200 nl/side) bilaterally was injected into PZ in C57BL/6J mice and implanted optical fibers on top. After virus injection, mice were allowed 3 weeks to recover. The optical fiber was connected to a 635 nm red laser source (inper, China) using an optical fiber sleeve. Each mouse was stimulated during different vigilant states for at least 8 trials per state in IP. Each stimulation was performed with a time interval for at least 3 min to ensure a full recovery for astrocytes. Mice with missed optical fiber placements were excluded. The control group expressing only mCherry underwent the same procedure and received the same intensity of laser stimulation.

### Drug Administration

NECA, CCPA, CPT, NBTI, and ARL67156 trisodium salt hydrate were from Sigma–Aldrich and CGS‐21680 hydrochloride was from Tocris. NECA (5 mm for rats, 0.25 mm for mice), CCPA (2 mm), CPT (1 mm), and CGS‐21680 (4 mm) were made up to stock solution in dimethyl sulfoxide (DMSO) and then diluted to their final concentrations in sterile 0.9% saline. ARL67156 (2 mm) and NBTI (250 µm) were directly dissolved in sterile 0.9% saline and diluted to their final concentrations in sterile 0.9% saline. Intracerebral drug delivery was through a previously implanted infusion canula. When delivering drug, the internal core which protruded 2 mm beyond the ends of the guide canula were pulled out, and drugs (1 µL per side for rats, 0.3 µL per side for mice) were infused bilaterally. The drug delivery time was controlled in 3 min and the catheter was kept still for 10 min to minimize the upward flow of solution when pulling out. The vehicle control groups were given an equivalent amount of DMSO dissolved in sterile 0.9% saline or sterile 0.9% saline. Drug and vehicle‐administered animals are both included in one experiment. Animals have adapted to handling and the environment in the recording chamber a week before the experiment. Sleep recording was performed in 5 h after drug administration.

For drug administration coupled with optogenetics experiments, AAV‐GfaABC1D‐ChrimsonR‐mCherry (200 nl per side) bilaterally was injected into PZ in C57BL/6J mice and implanted double guide canula (I.D. 450 µm) on top. After surgery, mice were allowed 3 weeks to recover. The optical fiber was connected to a 635 nm red laser source (inper, China) using an optical fiber sleeve. After drug administration, the optic fiber patch cord was inserted into the canula. Optogenetic stimulation was performed in half an hour after drug delivery. Each mouse was stimulated during different vigilant states for at least 8 trials per state in IP.

### Immunohistochemistry and Imaging

All animals were anesthetized with 1% sodium pentobarbital and then perfused transcardially with 0.9% NaCl followed by 4% paraformaldehyde (PFA, wt/vol) dissolved in phosphate‐buffered saline (PBS, pH 7.4). The brains were removed and postfixed in 4% PFA at 4 °C overnight, then dehydrated in 30% sucrose (wt/vol) for 3–4 days at 4 °C. Coronal sections (30–40 µm) were cut on a microtome (CM 1950, Leica, Germany) and stored in PBS at 4 °C for further use. For immunostaining, each section was treated with cold methyl alcohol for 10 min. After washing with PBS, the sections were blocked in 10% bovine serum albumin (BSA, wt/vol) with 5% donkey serum (wt/vol) for 1.5 hr at room temperature and then incubated with primary antibody (rabbit anti‐GFAP 1:1000, Millipore; mouse anti‐NeuN, 1:500, Millipore; rabbit anti‐A_1_ receptor 1:1000, abcam; rabbit anti‐TUJ1 1:1500, Sigma; rabbit anti‐S100β 1:500, abcam) diluted in 5% BSA (wt/vol) at 4 °C for 24 h. The sections were then washed three times (10 min each) in PBS, followed by incubation with secondary antibody (Alexa Fluor 647 Donkey anti‐rabbit 1:1000, Invitrogen; Alexa Fluor 647 Donkey anti‐mouse 1:1000, Invitrogen; Alexa Fluor 546 Donkey anti‐mouse 1:1000, Invitrogen; Alexa Fluor 546 Donkey anti‐rabbit 1:1000, Invitrogen; Alexa Fluor 488 Donkey anti‐mouse 1:1000, Invitrogen; Alexa Fluor 488 Donkey anti‐rabbit, Invitrogen) for 2 h at room temperature. Then, they were incubated for 5 min with DAPI and washed 3 times with PBS. Finally, the sections were mounted on microscope slides. Fluorescence images were captured with Olympus FV‐1200 and Olympus FV‐3000 (Japan) confocal microscopes.

### RNAscope In Situ Hybridization

RNAscope Fluorescent Multiplex Assays with the Slc32a1 (Vgat), Slc17a6 (VgluT2), and A2AR probes were used to determine the distribution of adenosine receptors in the PZ. Brain sections (40 µm thick) from C57BL/6J mice, containing PZ region, were collected with a cryostat (Leica CM 1950) and stored in DepC‐PBS. Floating sections were mounted on glass slides, heated at 60 °C for 2 h, and stored at −80 °C until further use. Slides were treated with ethanol 3 times (5 min each) and air‐dried at room temperature. Then, the slides were pretreated with hydrogen peroxide at room temperature for 10 min and washed twice in DepC‐PBS (2 min each). Protease digestion was performed in a 40 °C HybEZ oven for 15 min. After a 3‐min wash in DepC‐PBS and a rinse in DepC‐ddH2O, slides were hybridized with pre‐warmed Slc32a1 (Vgat), Slc17a6 (VgluT2), or A2AR probes in a 40 °C HybEZ oven for 2 h. Signal amplification was performed using a TSA‐based fluorescent label. The RNAscope Multiplex Fluorescent Reagent Kit v2 and specially designed probes (ACDBio Inc.) were used. The probes were alternated across all sections to ensure that both posterior and anterior sections from the PZ were analyzed with each probe type.

### Cell Culture

Primary brainstem and PZ cell cultures were prepared as described previously with some modifications.^[^ ^62^ ^]^ The brainstem or PZ region of GFAP‐ChR2‐EYFP rats on postnatal day 0 (P0) were dissected and dissociated by 0.25% trypsin. Cells were repopulated in Dulbecco's modified Eagle's medium (DMEM) culture medium containing 10% fetal bovine serum and 10% Ham's F‐12 (all from Gibco) at a cell density of 20000 /ml. The neurons were cultivated on a layer of astrocytes and maintained at 37 °C in a 5% CO_2_ incubator. The culture medium was changed every 2–3 days. 14 days later, the cocultured cells were used for immunostaining and fluorescence image acquisition.

### Statistical Analysis

All animals were randomly assigned to treatment groups and all data were randomly collected. The data values were first tested if they came from a normally distributed population with the D'Agostino–Pearson omnibus, Shapiro–Wilk, and Kolmogorov–Smirnov normality tests. Two‐tailed paired or unpaired t‐tests, Wilcoxon rank tests, one‐way or two‐way ANOVA tests followed by Tukey's, Bonferroni's, or Sidak's multiple comparisons tests, the Friedman test, or the Kruskal–Wallis test followed by Dunn's multiple comparisons test were used. Pearson correlation analyses were performed in fiber photometry experiments as indicated in the figure legends. Statistical significance was defined as *p* ≤ 0.05. All statistical analyses were performed with GraphPad Prism (Version 8.2.1) or MATLAB.

## Conflict of Interest

Authors declare that they have no competing interests.

## Author Contributions

Y.Z., J.M., and Y.L. contributed equally to this work, with Y.Z., Y.Q.Y., and S.D. responsible for conceptualization and methodology; Y.Z. and J.M. handling software; Y.Z., J.M., Y.L., M.G., L.T., Y.S., and X.F. involved in investigation; Y.Z., J.M., Y.L., M.G., and L.T. focusing on visualization; Y.Z., Y.Q.Y., and S.D. writing the original draft; Y.Z., J.M., Y.Q.Y., and S.D. conducting review and editing; Y.Q.Y. and S.D. managing funding acquisition; H.F.L., L.S., Y.L., L.H.Z., Z.Q., X.M.L., Y.Q.Y., and S.D. providing resources; and Y.Q.Y. and S.D. overseeing the project.

## Supporting information



Supporting Information

## Data Availability

The data that support the findings of this study are available from the corresponding author upon reasonable request.
