# Supplementary material for: Adenosine‐Dependent Arousal Induced by Astrocytes in a Brainstem Circuit
Source: Adv Sci (Weinh). 2024 Nov 4;11(48):2407706. doi: 10.1002/advs.202407706 (PMC11672293; doi:10.1002/advs.202407706)
Supplement: Supplementary file 1 — Supporting Information [file ADVS-11-2407706-s001.docx]

Supporting Information

**Adenosine-Dependent Arousal Induced by Astrocytes in a Brainstem Circuit**

*Yuwei Zhu, Jiale Ma, Yulan Li, Mengyang Gu, Xiang Feng, Yujin Shao, Lei Tan, Hui-fang Lou, Li Sun, Yijun Liu, Ling-hui Zeng, Zilong Qiu, Xiao-ming Li, Shumin Duan,* and Yan-qin Yu**


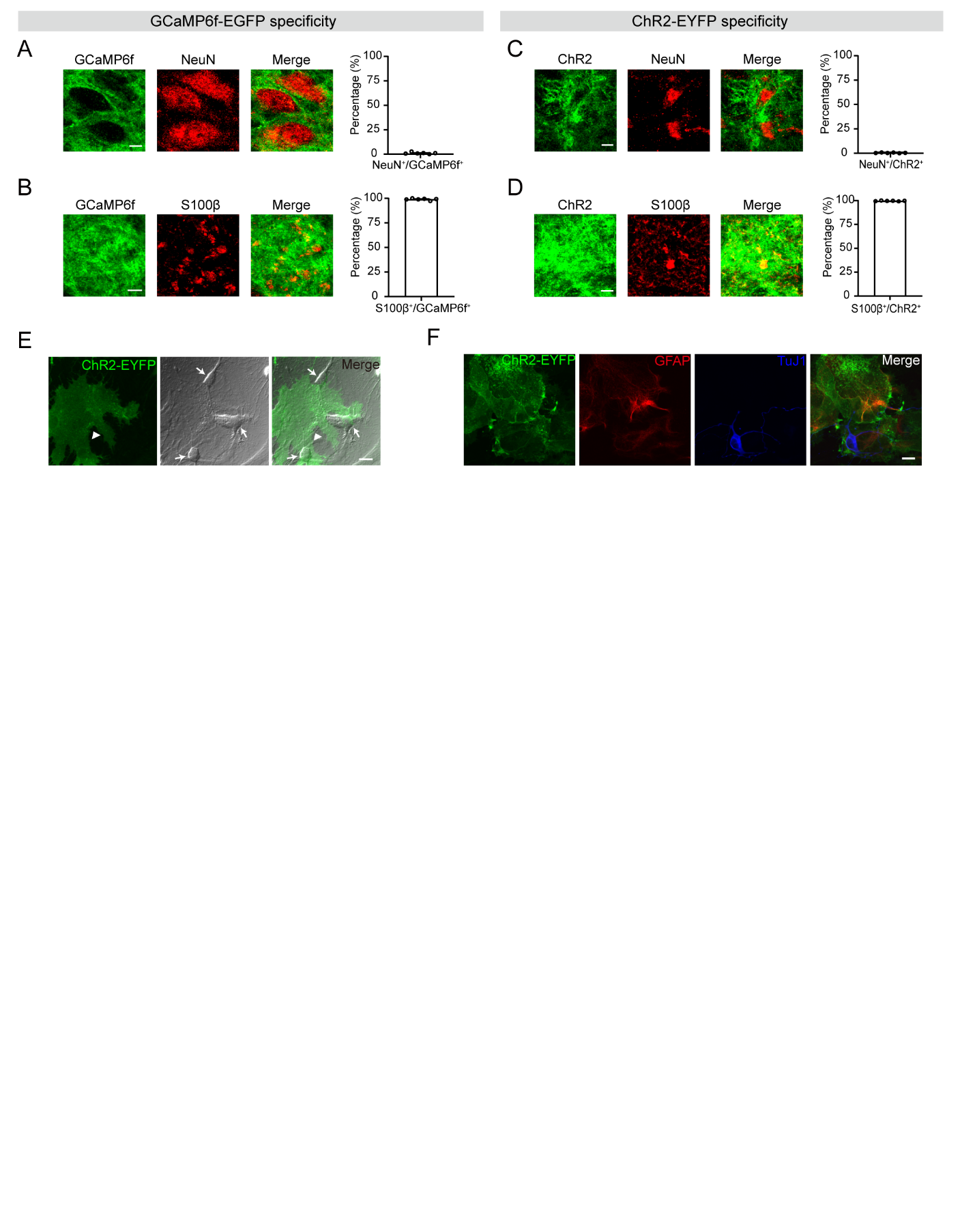
 **Figure S1.** The specificity of astrocyte-specific AAV-encoded GCaMP6f and GFAP-ChR2-EYFP rats. Related to Figure 1 and 2.

A-B) Left: Representative images of GCaMP6f expression in the PZ labelling with NeuN (A) and S100β (B). Scale bar, 10 µm; green, GCaMP6f; red, NeuN or S100β. Right: percentage of GCaMP6f-expressing neurons (A) and astrocytes (B). n = 6. Data are presented as the mean ± SEM.

C-D) Left: Representative images of ChR2 expression in the PZ labelling with NeuN (C) and S100β (D). Scale bar, 10 µm; green, ChR2; red, NeuN or S100β. Right: percentage of ChR2-expressing neurons (C) and astrocytes (D). n = 6. Data are presented as the mean ± SEM.

E) Representative images of cultured cells from the brainstem of GFAP-ChR2-EYFP rats that ChR2-EYFP is solely expressed within astrocytes and not in neurons. Scale bar, 50 µm; green, ChR2-EYFP. White triangle, astrocyte; white arrow, neuron.

F) Representative images of cultured cells in the PZ from GFAP-ChR2-EYFP rats that the expression of ChR2-EYFP was colocalized with GFAP while not with TUJ1. Scale bar, 50 µm; green, ChR2-EYFP; red, GFAP; blue, TUJ1.


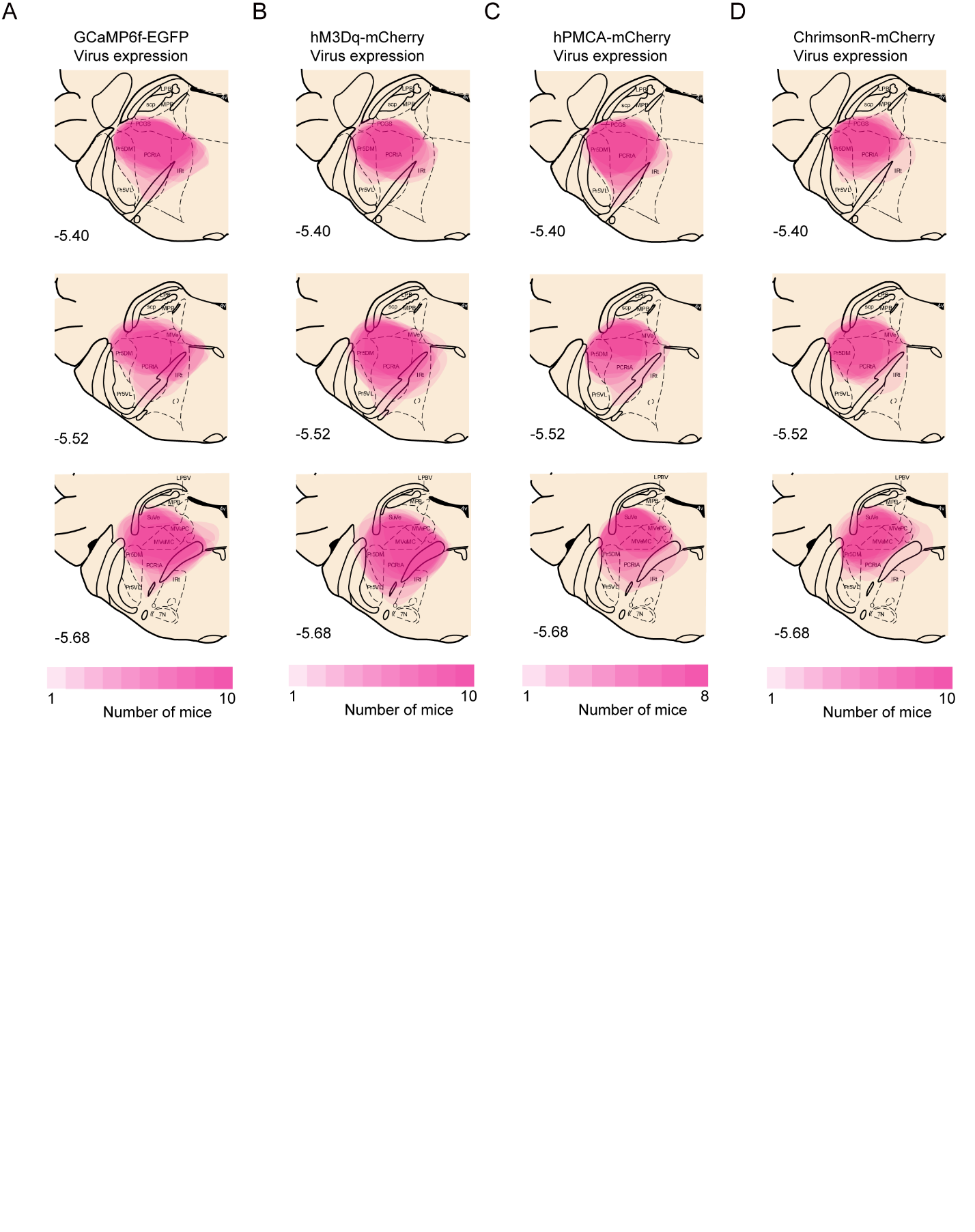


**Figure S2.** Virus expression range of astrocyte labelling AAV viruses. Related to Figure 1, 2, 3, and 5.

A-D) Overlay of GCaMP6f-EGFP (A), hM3Dq-mCherry (B), hPMCA-mCherry (C) ChrimsonR-mCherry (D), expression in the PZ.


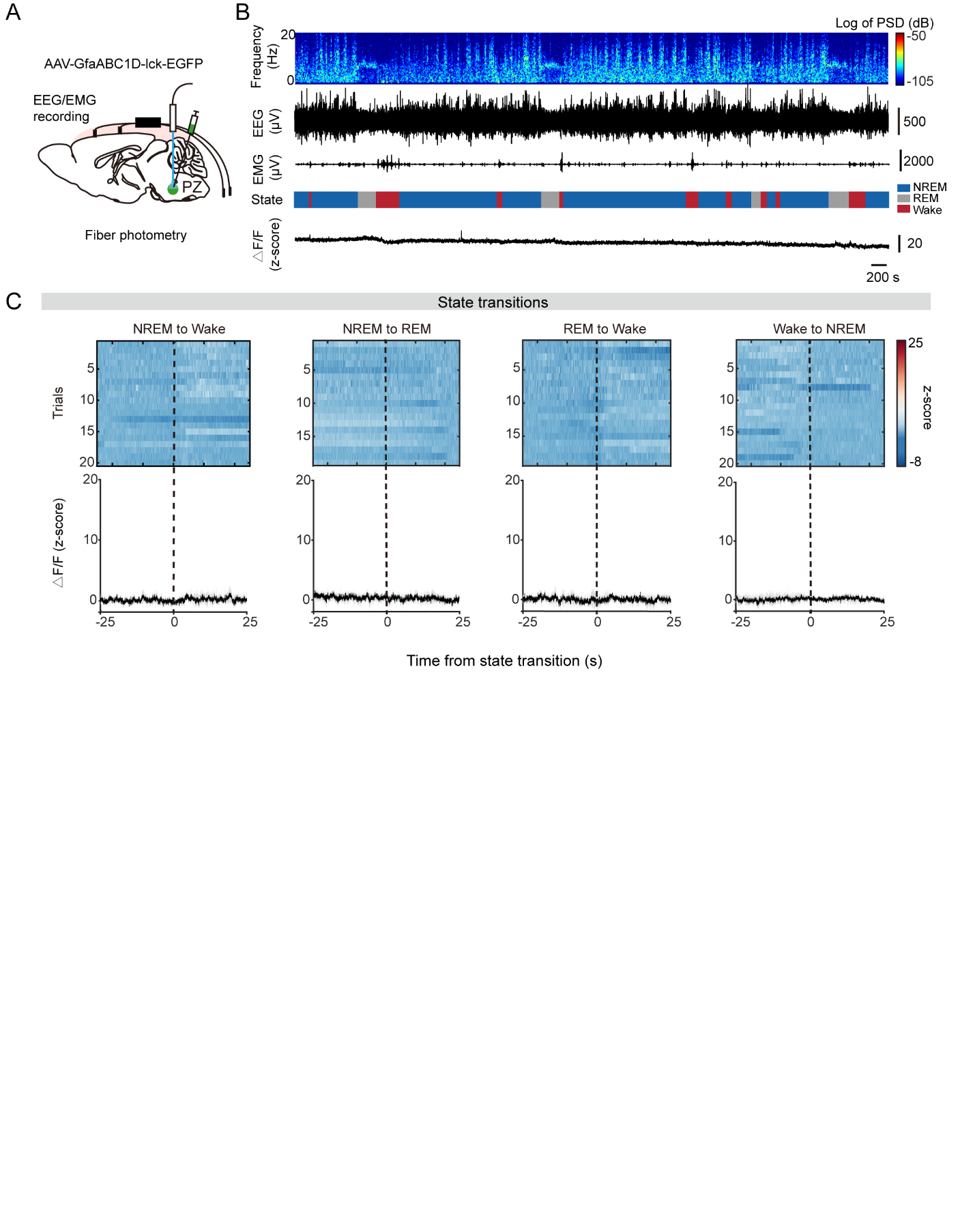


**Figure S3.** The EGFP fluorescence does not significantly fluctuate throughout sleep-wake cycles. Related to Figure 1.

A) Setup for fiber photometric recording of AAV-GfaABC1D-EGFP fluorescence in the PZ in combination with EEG/EMG signals across sleep-wake cycles.

B) Top to bottom, EEG power spectrogram (0-20 Hz), EEG traces, EMG traces, vigilant states (color coded) and EGFP fluorescence traces (z-score).

C) Heatmaps show EGFP fluorescence traces during the 25 s before and after state transitions between NREM sleep, REM sleep, and wakefulness (top). Line plots are mean ΔF/F (±s.e.m.) during state transitions under baseline conditions (bottom). Vertical dashed lines indicate time of state transitions. *n =* 4 mice. Data are presented as the mean ± s.e.m..


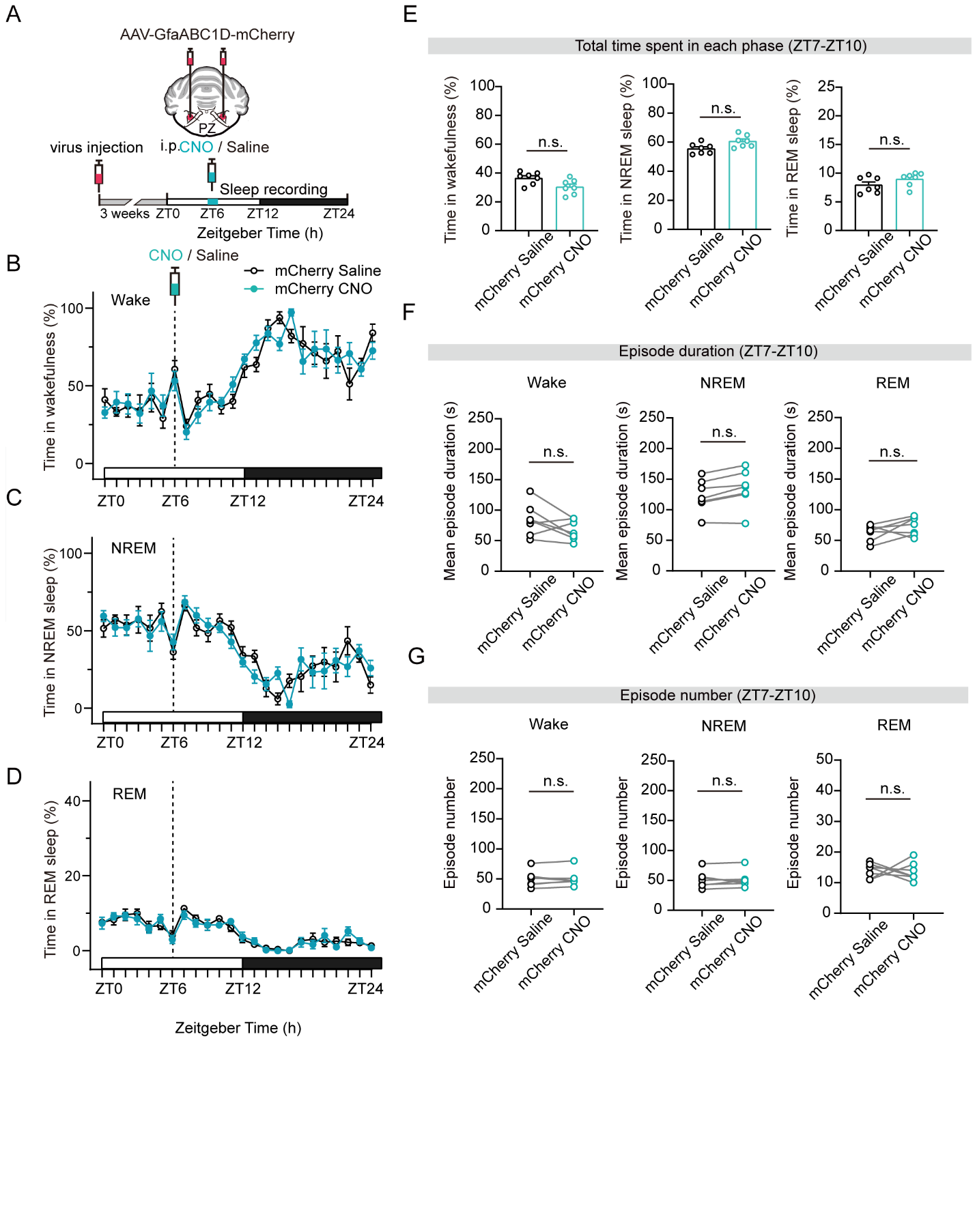


**Figure S4.** The dosage of CNO does not exert a significant impact on sleep-wake cycles. Related to Figure 2.

A) Setup for bilateral injection of AAV-GfaABC1D-mCherry virus into the PZ followed by intraperitoneal injection of CNO or saline at ZT6 after a 3-week expression period.

B-D) Hourly percentages (±s.e.m.) of wakefulness (B), NREM sleep (C), and REM sleep (D) of mCherry Saline group (*n =* 7 mice) and mCherry CNO group (*n =* 7 mice) throughout ZT0-ZT24 period. Two-way ANOVA test, Sidak's multiple comparisons test.

E) Time spent in wakefulness, NREM sleep, and REM sleep between mCherry Saline group (*n =* 7 mice) and mCherry CNO group (*n =* 7 mice) in 3 h after CNO injection (ZT7-ZT10). A two-tailed paired t test. n.s. indicates not statistically significant.

F) Mean episode duration of each state between mCherry Saline group (*n =* 7 mice) and mCherry CNO group (*n =* 7 mice) in 3 h after CNO injection (ZT7-ZT10). A two-tailed paired t test.

G) Episode number of each state in 3 h after CNO injection (ZT7-ZT10) between mCherry Saline group (*n =* 7 mice) and mCherry CNO group (*n =* 7 mice). Wake: a Wilcoxon matched-pairs signed rank test; NREM: a Wilcoxon matched-pairs signed rank test; REM: a two-tailed paired t test.


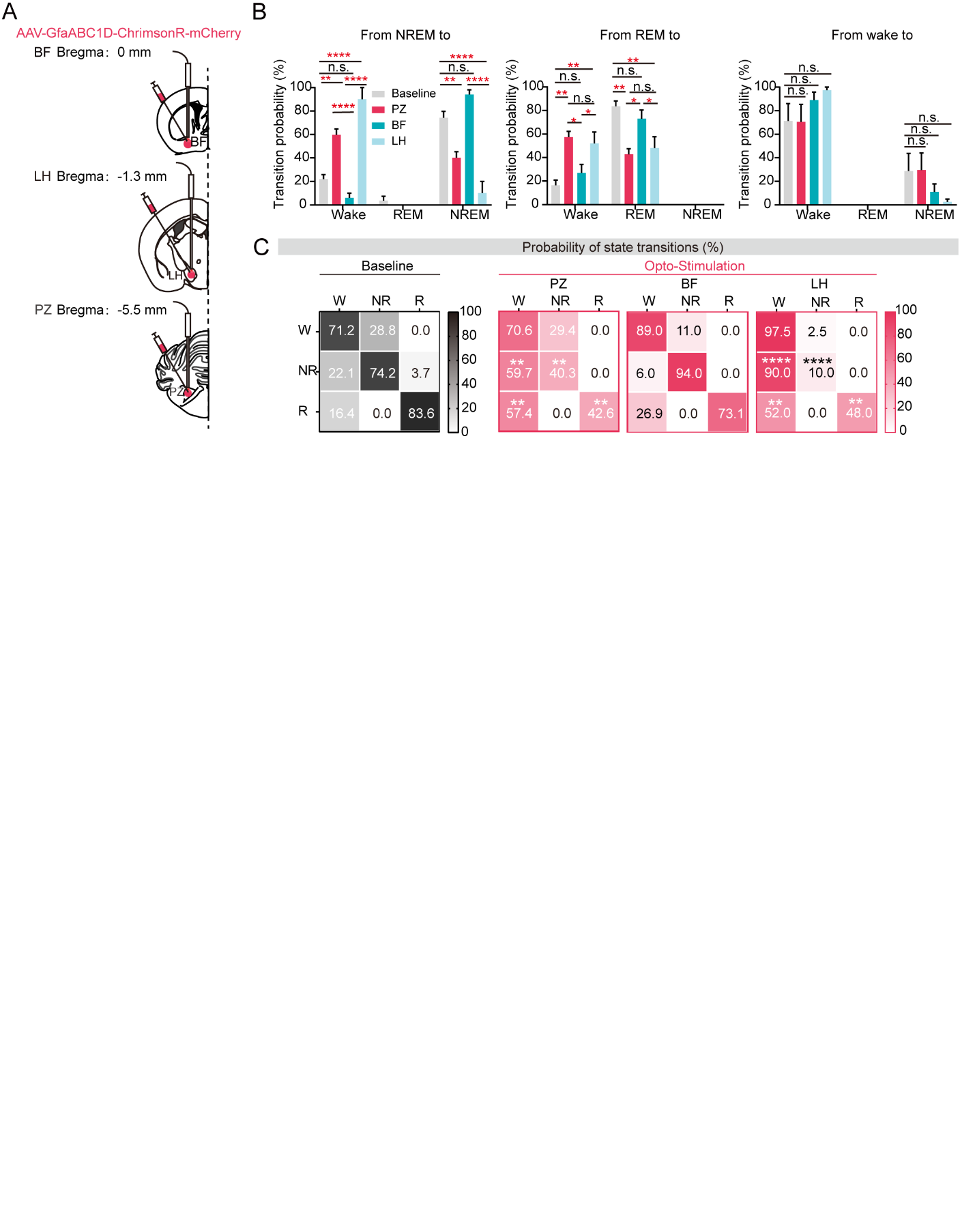


**Figure S5.** Region-dependent effects of astrocytic activation on sleep-wake transitions. Related to Figure 2.

A) Setup for comparing the effects of astrocyte activation in the BF, LH, and PZ, through respective injections of AAV-GfaABC1D-ChrimsonR-mCherry virus unilaterally.

B) Probability of state transitions by optogenetic activation of astrocytes in the PZ, BF and LH. Two-way ANOVA test, Tukey's multiple comparisons test. From NREM to Wake: *****P* < 0.0001, ***P* = 0.0028; From NREM to NREM: *****P* < 0.0001, ***P* = 0.0079. From REM to Wake and From REM to REM: ***P* = 0.0032 (Baseline vs. PZ), ***P* = 0.0044 (Baseline vs. LH), **P* = 0.0174 (PZ vs. BF), **P* = 0.0258 (BF vs. LH).

C) Probability of state transitions without (*n =* 3 mice) or with laser stimulation in the PZ (*n =* 3 mice), BF (*n =* 5 mice), and LH (*n =* 5 mice). Numbers in heatmap indicate averaged transition probability (%) from left to upper state. Color bar shows percentage. Two-way ANOVA test, Tukey's multiple comparisons test. PZ, ***P* = 0.0028 (NREM to Wake), ***P* = 0.0079 (NREM to NREM), ***P* = 0.0032 (REM to Wake and REM to REM); LH, *****P* < 0.0001 (NREM to Wake and NREM to NREM), ***P* = 0.0044 (REM to Wake and REM to REM).


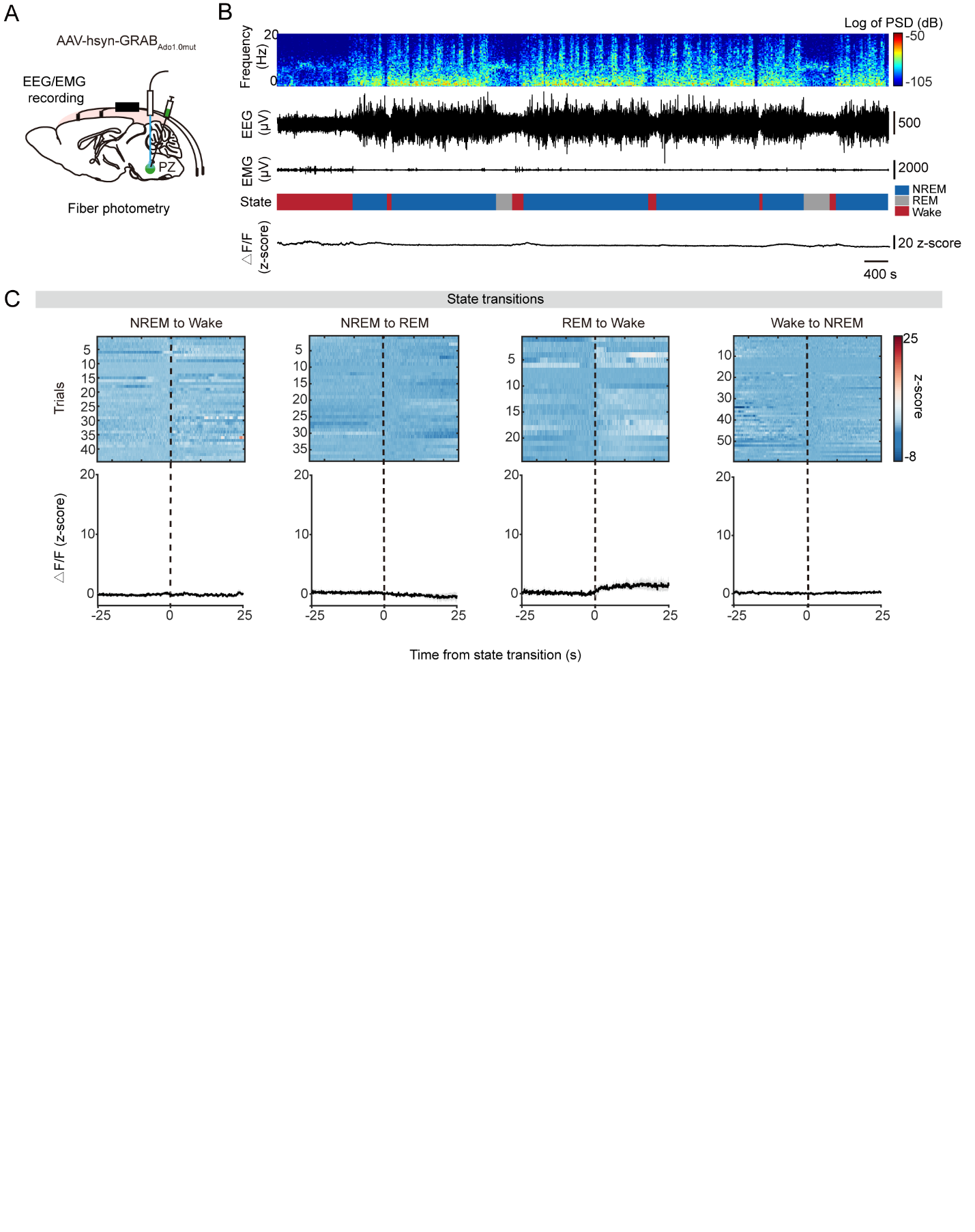


**Figure S6.** The GRAB_Ado-mut._ fluorescence does not significantly fluctuate throughout sleep-wake cycles. Related to Figure 4.

A) Setup for fiber photometric recording of GRAB_Ado-mut._ fluorescence in the PZ in combination with EEG/EMG signals across sleep-wake cycles.

B) Top to bottom, EEG power spectrogram (0-20 Hz), EEG traces, EMG traces, vigilant states (color coded) and GRAB_Ado-mut._ fluorescence traces (z-score).

C) Heatmaps show GRAB_Ado-mut._ fluorescence traces during the 25 s before and after transitions between NREM sleep, REM sleep, and wakefulness (top). Line plots are mean ΔF/F (±s.e.m.) during state transitions under baseline conditions (bottom). Vertical, dashed lines indicate time of state transitions. *n =* 6 mice.


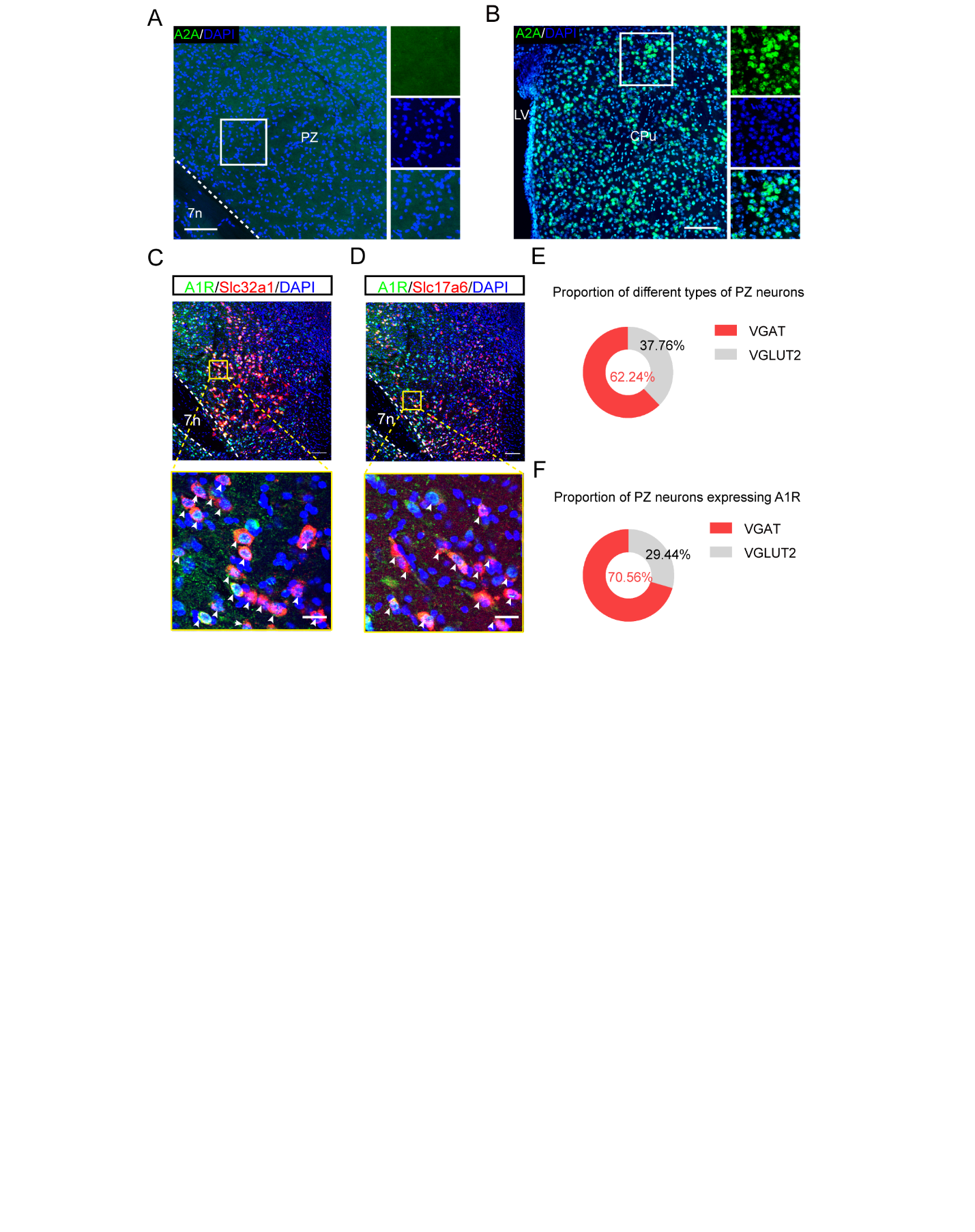


**Figure S7.** The distribution of adenosine receptors in the PZ region. Related to Figure 5.

A-B) Representative images of A_2A_ receptor expression in the PZ (A) and CPu (B) using RNAscope in situ hybridization staining of A_2A_ receptors (green) and DAPI (blue), with zoomed-in graphs on the right. Scale bar, 100µm.

C-D) Top: Representative images of A1 receptor colabelling with Slc32a1(Vgat) (C) or Slc17a6 (Vglut) (D) in the PZ. Scale bar, 50 µm; green, A1R; red, Slc32a1or Slc17a6; blue, DAPI. Bottom: Magnified views of the boxed areas from the top panel; scale bar, 10 μm.

E) Pie chart showing the proportion of Vgat- and Vglut- positive neurons in the PZ. n = 9.

F) Pie chart showing the proportion of A1R-expressing neurons that are Vgat- and Vglut- positive in the PZ. n = 9.


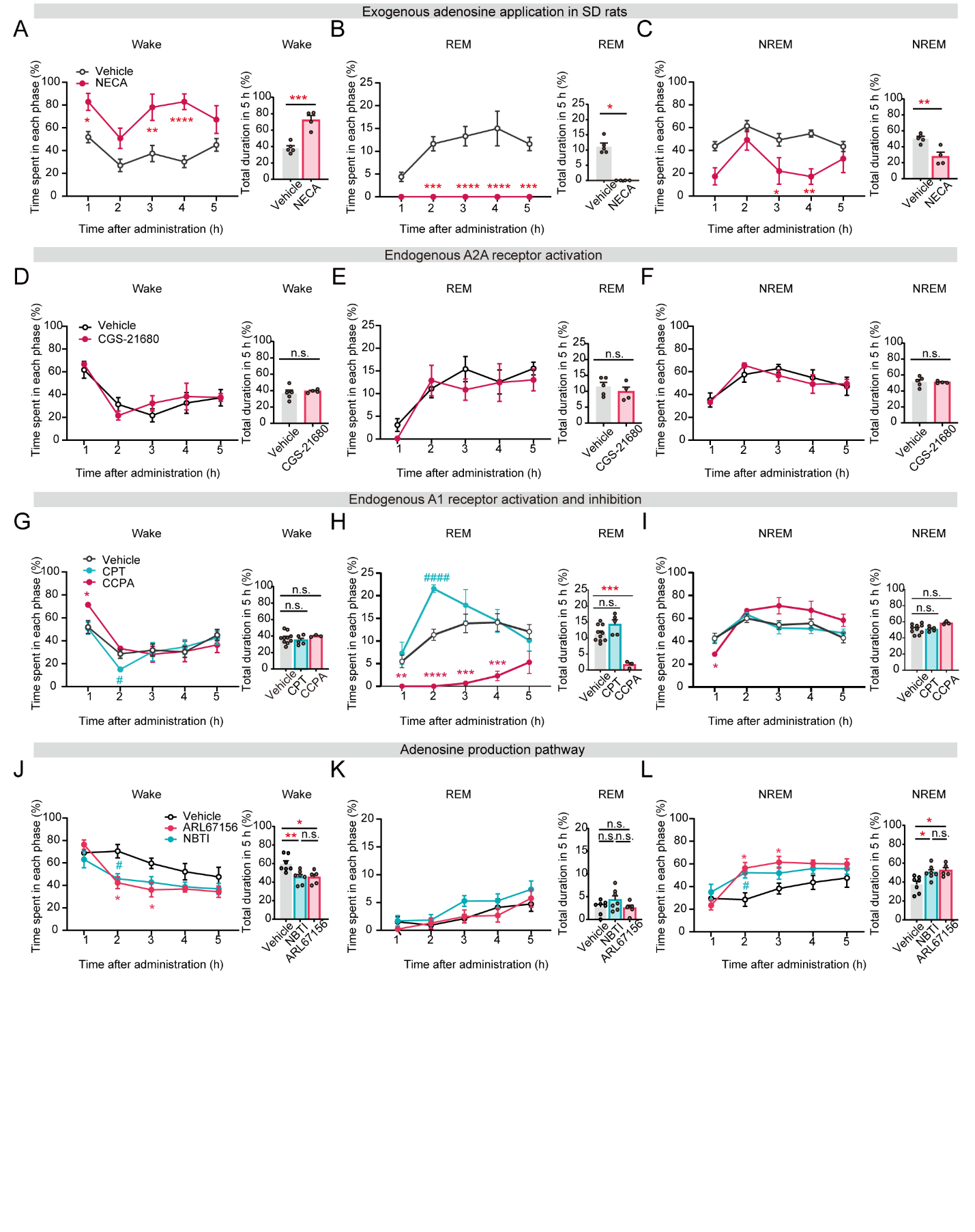


**Figure S8.** Adenosine plays a central role in promoting wakefulness by acting on A_1_ instead of A_2A_ receptors. Related to Figure 5.

A-C) Left, hourly percentages of time spent in wakefulness (A), REM sleep (B), and NREM sleep (C) in 5 h after administration of NECA (*n =* 4 rats) or vehicle (*n =* 5 rats) in the PZ of SD rats. Two-way ANOVA test, Sidak's multiple comparisons test. Wake: **P* = 0.0313, ***P* = 0.0027, *****P* < 0.0001; REM: ****P* = 0.0002, *****P* < 0.0001; NREM: **P* = 0.0436, ***P* = 0.0025. Right, percentage of total duration spent in each phase within 5 h after administration of NECA or vehicle. Wake: a two-tailed unpaired t test ****P* = 0.0006; REM: Kolmogorov-Smirnov test **P* = 0.0159; NREM: a two-tailed unpaired t test ***P* = 0.0047.

D-F) Left, hourly percentages of time spent in wakefulness (D), REM sleep (E), and NREM sleep (F) in 5 h after administration of CGS-21680 (A_2A_ receptor agonist, *n =* 4 rats) or vehicle (*n =* 5 rats) in the PZ of SD rats. Two-way ANOVA test, Sidak's multiple comparisons test. Right, percentage of total duration spent in each phase within 5 h after administration of CGS-21680 or vehicle. Wake, NREM: a two-tailed unpaired t test with Welch's correction; REM: a two-tailed unpaired t test.

G-I) Left, hourly percentages of time spent in wakefulness (G), REM sleep (H), and NREM sleep (I) in 5 h after administration of CCPA (A_1_ receptor agonist, *n =* 3 rats), CPT (A_1_ receptor inhibitor, *n =* 5 rats), or vehicle (*n =* 10 rats) in the PZ of SD rats. Two-way ANOVA test, Tukey's multiple comparisons test. Wake: Vehicle vs. CCPA **P* = 0.0288 (1 h); Vehicle vs. CPT ^#^*P* = 0.0320 (2 h); REM: Vehicle vs. CCPA, ***P* = 0.0080 (1 h), *****P* < 0.0001 (2 h), ****P* = 0.0003 (3 h), ****P* = 0.0007 (4 h); Vehicle vs. CPT, ^####^*P* < 0.0001 (2 h); NREM: Vehicle vs. CCPA, **P* = 0.0498 (1 h). Right, percentage of total duration spent in each phase within 5 h after administration of CCPA, CPT or vehicle. An ordinary one-way ANOVA test, Tukey's multiple comparisons test, REM: Vehicle vs. CCPA, *** *P* = 0.0001.

J-L) Left, hourly percentages of time spent in wakefulness (J), REM sleep (K), and NREM sleep (L) in 5 h after administration of ARL67156 (CD73 inhibitor, *n =* 5 mice), NBTI (ENT inhibitor, *n =* 7 mice) or vehicle (*n =* 7 mice). Two-way ANOVA test, Tukey's multiple comparisons test, Wake: 2 h, ^#^*P* = 0.0190 (Vehicle vs. NBTI), **P* = 0.0146 (Vehicle vs. ARL67156); 3 h, **P* = 0.0387 (Vehicle vs. ARL67156). NREM: 2 h, ^#^*P* = 0.0216 (Vehicle vs. NBTI), **P* = 0.0120 (Vehicle vs. ARL67156); 3 h, **P* = 0.0232 (Vehicle vs. ARL67156). Right, percentage of total duration spent in each phase during 5 h following the administration of ARL67156 (*n =* 5 mice), NBTI (*n =* 7 mice) or vehicle (*n =* 7 mice). An ordinary one-way ANOVA test, Tukey's multiple comparisons test, Wake: ***P* = 0.0090 (Vehicle vs. NBTI), **P* = 0.0145 (Vehicle vs. ARL67156); NREM: **P* = 0.0174 (Vehicle vs. NBTI), **P* = 0.0115 (Vehicle vs. ARL67156).


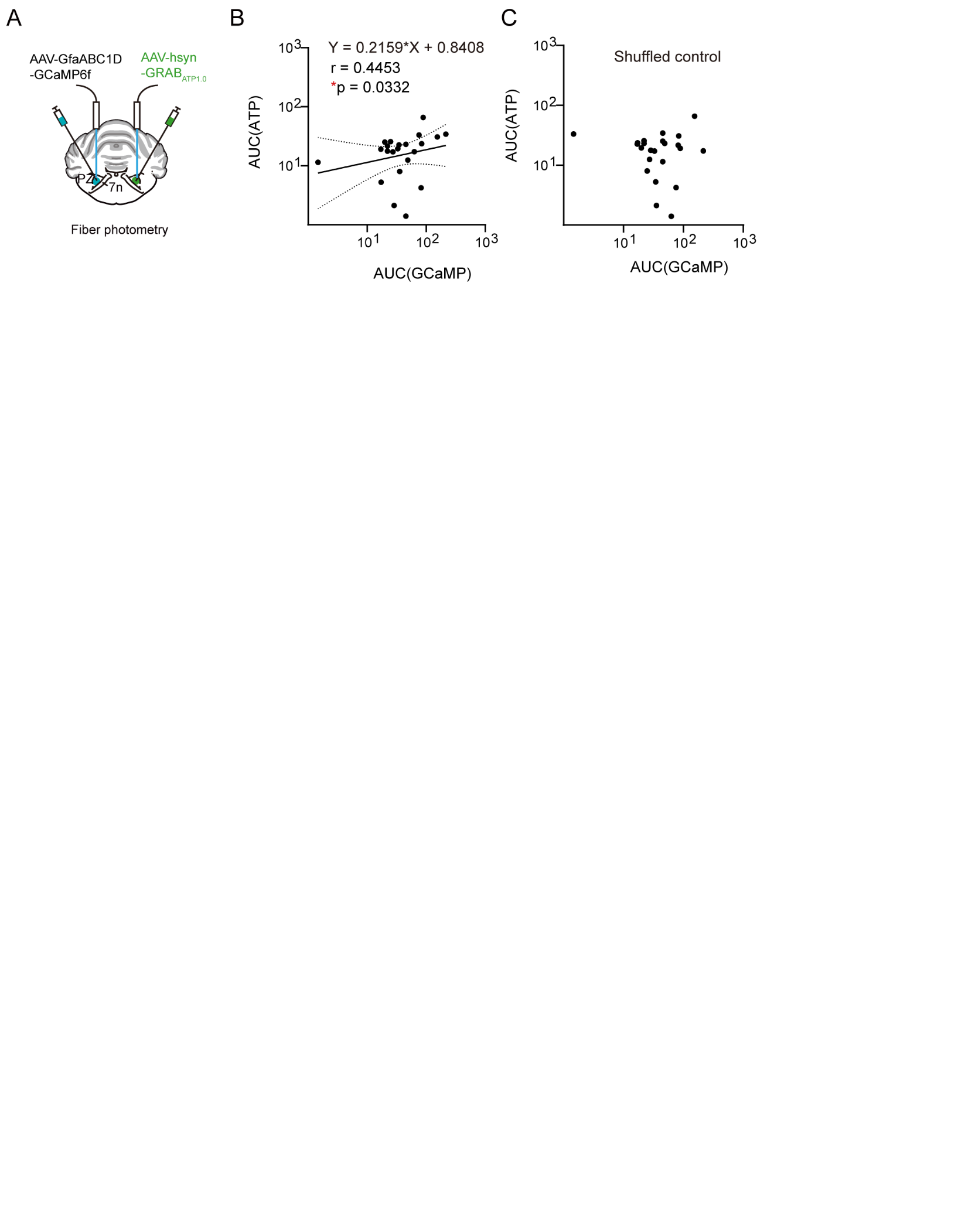


**Figure S9.** The correlation analysis of GCAMP and GRAB_ATP1.0_ signals. Related to Figure 5.

A) Setup for bilateral fiber photometric recording of ATP with GRAB_ATP1.0_ and astrocytic Ca^2+^ activity within the PZ during sleep-wake cycles.

B-C) Correlation analysis of GCaMP6f and GRAB_ATP1.0_ signals (B) and the shuffled control (C). n = 23 trials, recorded from 3 individuals. Pearson correlation analysis, **P* = 0.0332, r = 0.4453, line shows linear regression line (Y = 0.2159*X+0.8408), dot line indicates 95% regression range.


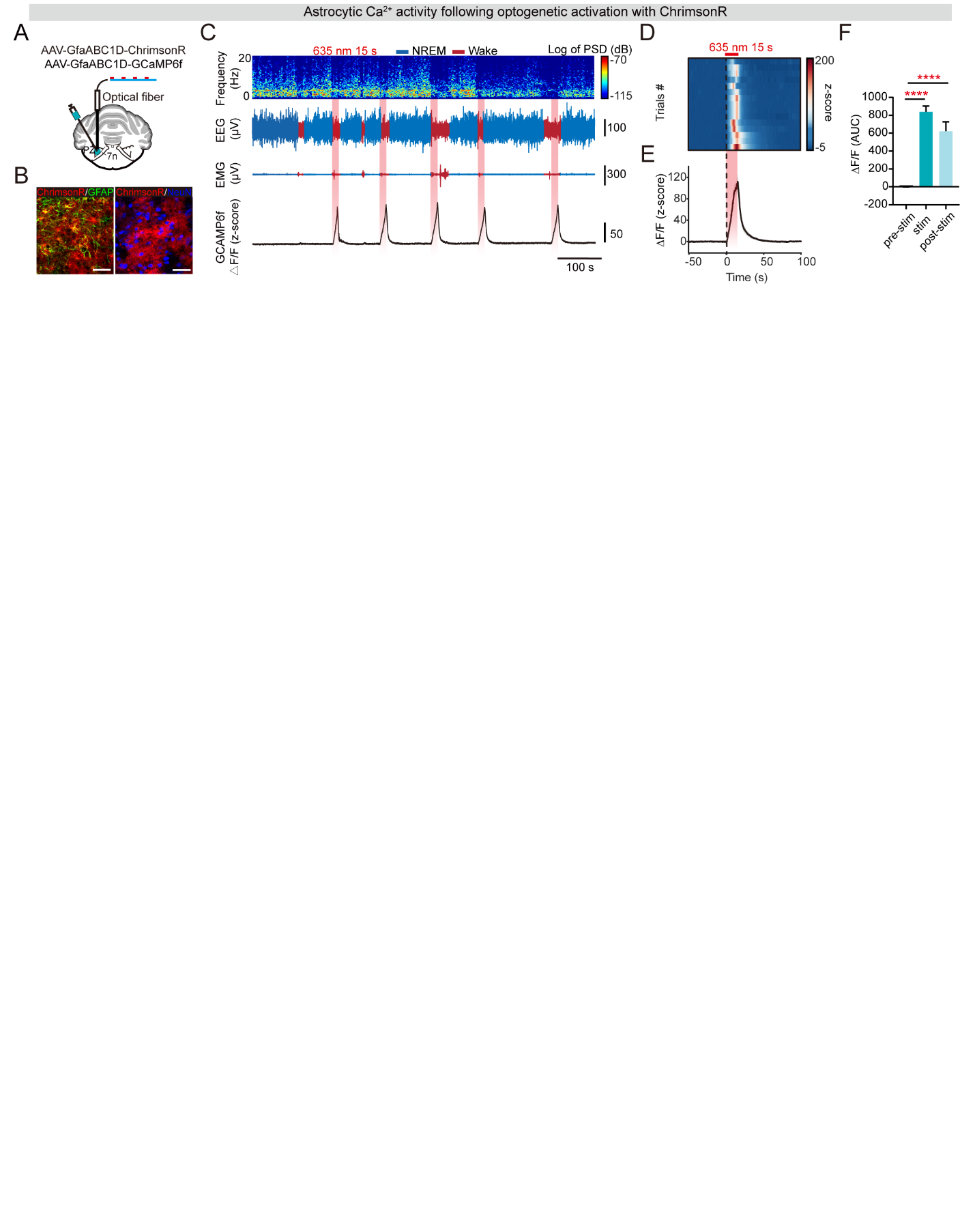


**Figure S10.** Optogenetic activation of astrocytes by ChrimsonR significantly increases astrocytic Ca^2+^ activity. Related to Figure 5.

A) Setup for fiber photometric recording of astrocytic Ca^2+^ activity in the PZ while optogenetically activating astrocytes by mixed injection of GCaMP6f with ChrimsonR virus.

B) Representative images of AAV-GfaABC1D-ChrimsonR-mCherry expression in the PZ co-localized with GFAP, while not with NeuN. Scale bar, 50 µm; red, ChrimsonR; green, GFAP; blue, NeuN.

C) Top to bottom, EEG power spectrogram (0-20 Hz), EEG traces, EMG traces and representative Ca^2+^ fluorescence traces (z-score) during sleep-wake cycles; color code indicates NREM sleep and wakefulness. Red shade indicates 15 s optogenetic stimulation.

D-E) Heatmap shows Ca^2+^ fluorescence traces during the 50 s before and 100 s after the optogenetic stimulation (D). Line plots are mean ΔF/F (±s.e.m.) under optogenetic stimulation (E). Vertical dashed lines indicate time of laser stimulation. *n =* 15 trials.

F) Area under curve (AUC) of Ca^2+^ fluorescence in 15 s of pre-, during and post-stimulation periods. *n =* 15 trials, an ordinary one-way ANOVA test, *****P* < 0.0001.


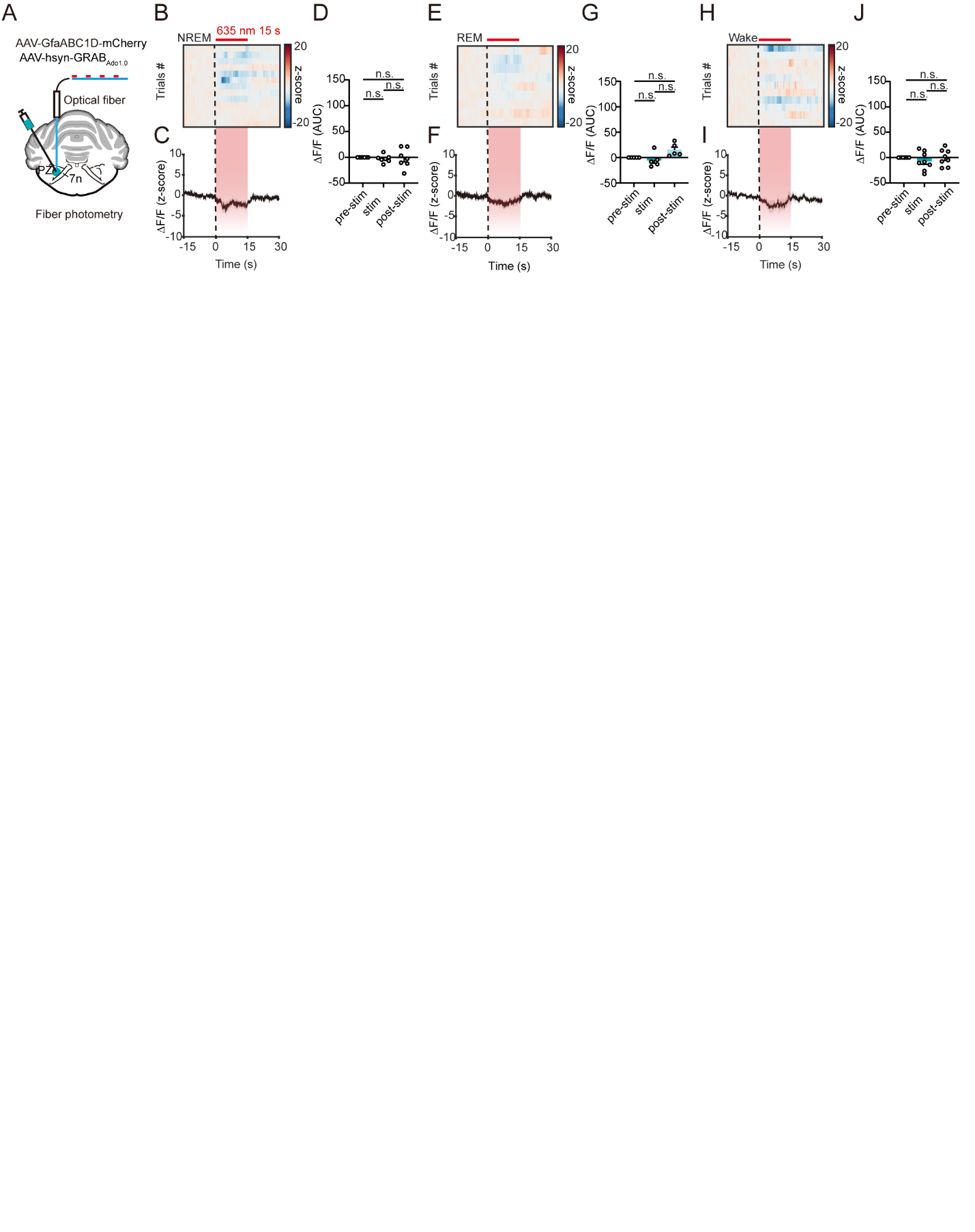


**Figure S11.** The laser itself does not result in a notable increase in GRAB_Ado1.0_ signals. Related to Figure 6.

A) Setup for fiber photometric recording of GRAB_Ado1.0_ signals in the PZ following laser stimulation by mixed injection of AAV-hsyn-GRAB_Ado1.0_ with AAV-GfaABC1D-mCherry virus.

B-J) Heatmaps show GRAB_Ado1.0_ fluorescence traces during the 15 s before and 30 s after optogenetic stimulation in NREM sleep (B), REM sleep (E), and wakefulness (H). Line plots are mean ΔF/F (±s.e.m.) under optogenetic stimulation in NREM sleep (C, *n =* 8 mice), REM sleep (F, *n =* 5 mice), and wakefulness (I, *n =* 5 mice). Area under curve (AUC) of GRAB_Ado1.0_ signals in 15 s of pre-, during and post-stimulation periods in NREM sleep (D, *n =* 7 mice), REM sleep (G, *n =* 5 mice), and wakefulness (J, *n =* 8 mice). A one-way ANOVA test, Tukey's multiple comparisons test.


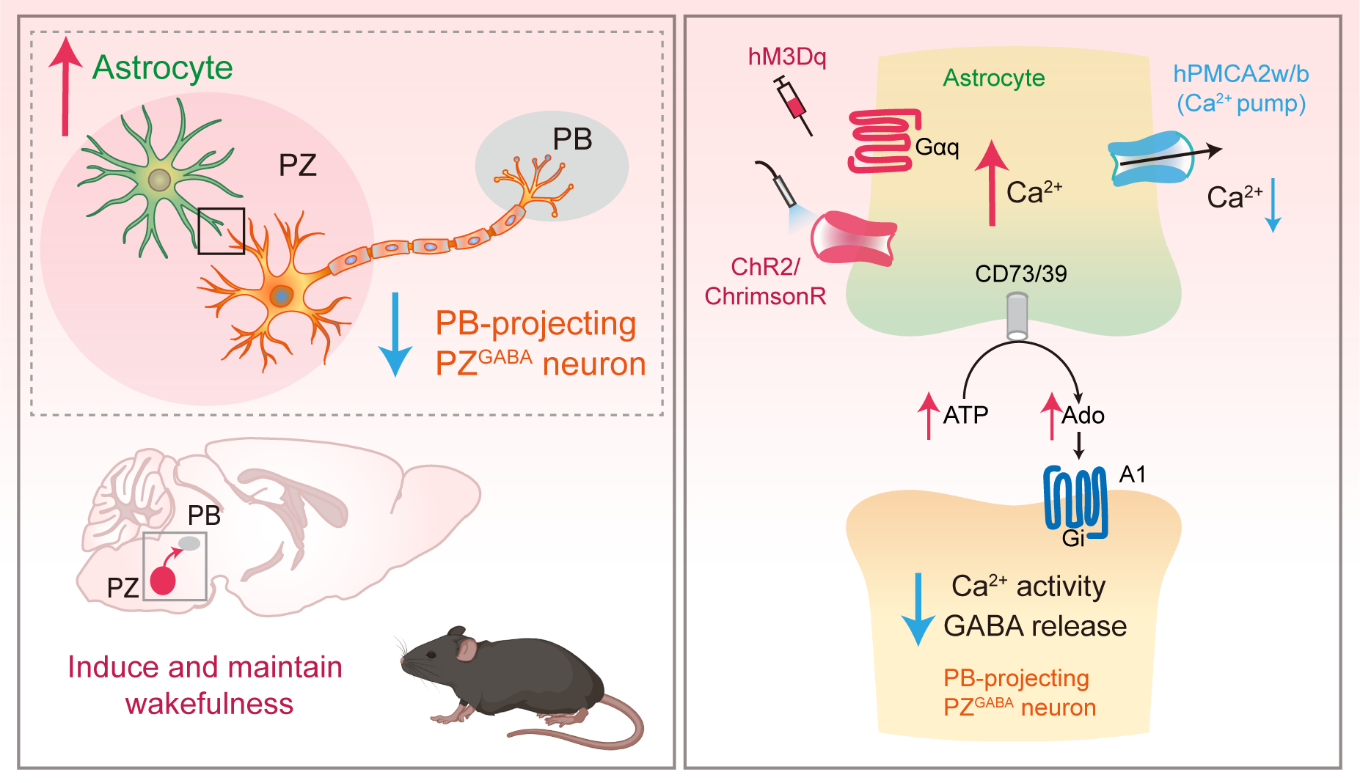


**Figure S12.** Summary of the molecular and neural circuit mechanisms underlying PZ astrocyte activation in sleep-wake regulation.
